# Genome-wide analysis of the COMT gene family in *Avena sativa*: insights into lignin biosynthesis and disease defense mechanisms

**DOI:** 10.3389/fpls.2025.1609698

**Published:** 2025-06-19

**Authors:** Yuanbo Pan, Kuiju Niu, Fangming Shen, Guiqin Zhao, Yuehua Zhang, Jikuan Chai, Zeliang Ju

**Affiliations:** ^1^ College of Pratacultural Science, Gansu Agricultural University, Lanzhou, Gansu, China; ^2^ National Center of Pratacultural Technology Innovation (under preparation), Inner Mongolia Pratacultural Technology Innovation Center Co. Ltd, Hohhot, Inner Mongolia, China; ^3^ Key Laboratory of Superior Forage Germplasm in the Qinghai-Tibetan Plateau, Academy of Animal Husbandry and Veterinary Sciences, Qinghai University, Xining, Qinghai, China

**Keywords:** caffeic acid o-methyltransferase, bioinformatics analysis, expression pattern, lignin biosynthesis, plant disease defense

## Abstract

Caffeic acid O-methyltransferase (COMT) is a multifunctional enzyme involved in lignin biosynthesis and plays an important role in various primary and secondary metabolic pathways, including the plant stress response. In this study, we identified 37 *AsCOMT* genes from the oat (*Avena sativa*) whole-genome database, which are distributed across 11 chromosomes. Phylogenetic analysis grouped these genes into two major subfamilies, indicating that they are highly conserved during evolution and share close relationships with *COMT* genes from *Zea mays* and *Oryza sativa*. Cis-acting elements analysis revealed a rich presence of regulatory motifs related to plant hormone signaling and stress responses. Expression profiling of different oat varieties infected with powdery mildew and leaf spot disease showed significant upregulation or downregulation of several *AsCOMT* genes (e.g., *AsCOMT14*, *AsCOMT22*, *AsCOMT24*, *AsCOMT27*). Moreover, disease-resistant oat varieties have higher lignin contents compared to susceptible varieties. Overexpression of *AsCOMT23* and *AsCOMT27* in tobacco leaves resulted in significantly increased lignin content, highlighting the potential of these genes in lignin biosynthesis. These results offer a preliminary exploration of the role of *AsCOMT* in both lignin synthesis and the plant stress response, laying the groundwork for further functional studies and potential applications in oat breeding.

## 1 Introduction

Oats (*Avena sativa* L.) are an important global food crop widely used in both the food and feed industries due to their high content of dietary fiber, antioxidants, and essential nutrients. As a key annual cereal and forage crop in the Poaceae family (Butt et al., 2008), oats have strong resistance, excellent quality, high yield, good palatability, and ease of harvest and processing, making them a globally cultivated crop (Cerecetto et al., 2024). Major oat producers include Russia, Canada, the United States, Australia, Germany, Finland, China, and other countries in the Northern Hemisphere (Achleitner et al., 2008). With the growth of the global population and an increasing demand for nutritious and healthy foods, oat production continues to rise annually (Stewart and McDougall, 2014). However, fungal diseases such as powdery mildew and leaf spot often significantly affect the yield and quality of oats, posing substantial challenges to global oat production. To address these challenges, developing disease-resistant oat varieties has become a key strategy for improving yields. In-depth research into the disease resistance mechanisms of oats, particularly the gene families involved in disease resistance, can offer new molecular targets for molecular breeding.

Lignin is a complex organic polymer found within the supporting tissues of vascular plants and some algae. It is an important component of plant cell walls (Mahmood et al., 2018), playing a crucial role in wood and bark, by imparting rigidity to plant cells and tissues while helping to resist biodegradation (Leisola et al., 2012). Lignin is vital for mechanical support and the defense responses of plants (Lebedev and Shestibratov, 2021). It is also involved in plant stress tolerance and increasing lignin content often enhances a plant’s resistance to lodging, disease, and abiotic stress (Fukushima and Hatfield, 2001). The lignin biosynthesis pathway plays a key role in a plant’s defense against pathogen invasion by strengthening the cell wall and preventing pathogen penetration (Riaz et al., 2023). Through transgenic technology or genetic breeding methods that upregulate the expression of key enzyme genes in the lignin synthesis pathway, the lignin content and stress resistance of plants can be significantly improved (Wang et al., 2022). This approach provides new opportunities and methods for breeding crops with enhanced stress resistance.

Caffeic acid O-methyltransferase (COMT) is a key enzyme present in both plants and animals. It belongs to the S-adenosylmethionine-dependent methyltransferase family and is involved in various primary and secondary metabolic pathways (Wu et al., 2013). The enzymes encoded by members of this gene family play a key role in lignin biosynthesis (Byeon et al., 2014; Do et al., 2007). The expression level and enzyme activity of the COMT gene directly influence the synthesis rate and composition of lignin, as it is primarily responsible for catalyzing the methylation reaction of caffeic acid (or similar substrates) (Peracchi et al., 2024). Studies suggest that by regulating the expression of the COMT gene, it is possible to influence the lignin content and structure within plants, thereby affecting their growth, development, and stress resistance. The COMT gene family plays an important role in the stress and disease resistance of various plants (Liang et al., 2022). However, research on the composition of the COMT gene family in oats and its role in responding to fungal diseases remains limited. Therefore, studying the COMT gene family not only enhances our understanding of plant growth, development, and stress resistance mechanisms but also has important implications for plant breeding and agricultural production.

In recent years, advancements in molecular biology and bioinformatics technologies have deepened research on the COMT gene family. This study aims to conduct a genome-wide identification and analysis of the COMT gene family in oats and explore its response patterns under the stress of powdery mildew and leaf spot disease. Our study involves systematic gene family analysis, evolutionary relationship studies, and gene expression pattern exploration. Additionally, we aim to measure changes in lignin content in oat leaves after pathogen infection, as well as in tobacco leaves after transient expression of the AsCOMT gene. This research is valuable for understanding how plants adapt to environmental changes, enhancing plant stress resistance, and optimizing plant germplasm resources. Specifically, in crops like oats, studying the COMT gene family will help reveal disease resistance mechanisms, providing new genetic resources and strategies for breeding disease-resistant crops.

## 2 Materials and methods

### 2.1 Materials

This study utilizes two oat cultivars, *sativa* cv. ForagePlus’ and ‘*Avena sativa* cv. Molasses’, which differ in their susceptibility to powdery mildew and leaf spot infection. Seeds of ‘ForagePlus’ and ‘Molasses’ were purchased from Beijing Rytway Seed Co., Ltd. (Beijing, China) and CLOVER (Beijing, China), respectively. The pathogenic fungi responsible for powdery mildew (*Blumeria graminis* f.sp. *avenae*) and leaf spot disease (*Drechslera avenacea*) were isolated and stored in our laboratory. RNA-Seq data were obtained from the Plant Genomics & Phenomics Research Data Repository (https://doi.org/10.5447/ipk/2022/2) to analyze expression patterns in different tissues or at different developmental periods in the same tissue.

### 2.2 Identification of *AsCOMT* genes

The protein sequences of *COMT* genes from *A.thaliana*, *O.sativa*, and *Z.mays* were directly downloaded from the NCBI database (https://www.ncbi.nlm.nih.gov/; accessed on 6 January 2024). The oat genome file was downloaded from the GrainGenes database (https://wheat.pw.usda.gov/GG3/content/avena-sang-download) (Kamal et al., 2022). To identify the oat *COMT* genes, the BLAST wrapper in TBtools 2.07 was used to align known *COMT* genes to the oat genome (Chen et al., 2023). Redundant alleles were filtered out from the initially identified gene candidates to obtain the final set of non-redundant gene sequences. The naming of the *AsCOMT* genes was based on the reconstructed evolutionary tree, with gene names assigned according to previously annotated *COMT* genes from closely related species.

### 2.3 Characterization of AsCOMT proteins, sequence structure and phylogenetic analyses

The physicochemical properties of the AsCOMT protein sequences, including the number of amino acids, molecular weight, theoretical isoelectric point, instability index, aliphatic index, and total average hydrophilicity, were analyzed using the ExPAsy-ProtParam tool (https://web.expasy.org/protparam/) (Jayhoon and Kumar, 2024). The online tool TMHMM-2.0 (https://services.healthtech.dtu.dk/services/TMHMM-2.0/) was used to predict transmembrane spiral structures (Hallgren et al., 2022). Subcellular localizations predictions were made using ProtComp v. 9.0 (http://www.softberry.com/) (Chou and Shen, 2010). The protein’s secondary structure was predicted using SOPMA (https://npsa-prabi.ibcp.fr/cgi-bin/), and tertiary structure predictions were generated using SWISS-MODEL (https://swissmodel.expasy.org/; accessed on 8 January 2024) (Waterhouse et al., 2018; Bienert et al., 2017; Guex et al., 2009; Studer et al., 2020; Bertoni et al., 2017).

Conserved motif analysis of the COMT amino acid sequences in oats was conducted using the MEME online tool (http://meme-suite.org/; accessed on 8 January 2024) (Ghorbel et al., 2023). Gene structure and motif distributions were visualized using TBtools 2.07 (Chen et al., 2020). A phylogenetic tree of the COMT family was constructed using the maximum likelihood method in MEGA 11.0, incorporating 37 oat sequences, 16 A*. thaliana* sequences, 10 *O. sativa* sequences, and 18 *Z. mays* sequences. The protein sequences used for these analyses can be found in **Additional file 2**.

### 2.4 Cis-acting elements, chromosome distributions, gene duplication, and collinearity analysis

The cis-acting elements in the putative promoter regions of the *AsCOMT* genes, defined as the 2,000 bp upstream of the genes, were predicted using PlantCARE (http://bioinformatics.psb.ugent.be/webtools/plantcare/html/) (Lescot et al., 2002), and the predicted cis-acting elements were visualized using TBtools 2.07 (Chen et al., 2020).

The chromosomal localizations of the *COMT* genes were visualized using TBtools 2.07 (Chen et al., 2020). For collinearity analysis, the genome sequences of *A. thaliana*, *O. sativa*, and *Z. mays*, as described above, were used, with Circos 0.69 and TBtools 2.07 employed for visualization (Krzywinski et al., 2009). Protein-protein interactions among the *COMT* proteins were predicted using the online tool string (https://cn.string-db.org), and the results were visualized accordingly (Franceschini et al., 2012).

### 2.5 Oat growth conditions and biotic stress treatments

The seeds of two oat cultivars, ‘ForagePlus’ and ‘Molasses’, were sown in plastic pots containing a mixed medium of soil and vermiculite (2:1). The growth chambers were set to a daytime temperature of 25 °C and a nighttime temperature of 20 °C, with a 14-hour light: 10-hour dark cycle. Three replicates were performed for each treatment (control and inoculated). Twenty days after planting, the plants were inoculated with *Blumeria graminis* f.sp. *avenae* or *Drechslera avenacea* using the spore suspension method. *Blumeria graminis* f.sp. *avenae* conidia were collected from infected wheat leaves with a fine camel hairbrush and suspended in sterile distilled water (containing 0.05% Tween 20) at a concentration of 10^5^ conidia/mL. The *Drechslera avenacea* spore suspension was prepared by cultivating the fungus on potato dextrose agar (PDA) plates at 25°C for 10–12 days until sporulation was abundant. The spores were harvested by flooding the plates with sterile distilled water containing 0.05% Tween 20 and gently scraping the surface with a sterile glass rod. The spore suspension was filtered through a double layer of cheesecloth to remove mycelial fragments and adjusted to a concentration of 10^5^ conidia/mL. The conidial suspension was then applied to the oat plants using a fine mist spray bottle, ensuring complete coverage of foliage. Inoculated plants were incubated at 25°C with 80–90% relative humidity for 48 hours to promote infection, followed by maintenance at 60% humidity. After this incubation period, plants were monitored for symptom development. Leaves were sampled at 1, 3, 6, 9, and 12 days post-inoculation, with leaves collected before incubation (0 days) serving as the control. The sampled leaves were immediately frozen in liquid nitrogen and stored at -80°C for later RNA extraction.

### 2.6 RNA extraction and real-time quantitative PCR analysis

RNA extraction was performed using the Total RNA Extraction Kit (DP419, Tiangen). cDNA was then synthesized using the PrimerScript™ RT Reagent Kit with gDNA Eraser (R047A, Takara).

Primers specific to the oat *AsCOMT* gene were designed using Primer BLAST (https://www.ncbi.nlm.nih.gov/tools/primer-blast/; accessed on 6 January 2024). The primer sequences are provided in **Additional file 1: Table S2**. Quantitative real-time PCR (qRT-PCR) was performed using the SuperReal PreMix Plus (SYBR Green) fluorescence quantification kit. The total reaction volume of 20 µL consisted of 5 µL of diluted template (20 µL of cDNA diluted 20-fold with 180 µL of ddH_2_О), 3 µL of ddH_2_O, 1 µL of upstream and 1 µL of downstream primers (10 µmol·L^-1^), and 10 µL of 2 × SuperReal PreMix Plus (SYBR Green). The qRT-PCR reaction was carried out on a LightCycler^®^96 instrument with the following procedure: initial denaturation at 95 °C for 15 minutes, followed by 40 cycles of denaturation at 95 °C for 10 seconds and annealing at 58°C for 30 seconds. Three biological replicates were performed for each treatment, with three technical replicates for each biological replicate. The relative gene expression level was calculated using the 2^-ΔΔ Ct^ method (Damgaard and Treebak, 2022), with *Actin* used as the reference gene.

### 2.7 Determination of lignin content and correlation of gene expression levels with lignin content

The lignin content was determined using the method outlined by Bhaskara Reddy et al. (1999). The Pearson correlation coefficient between *AsCOMT* gene expression and lignin content was calculated, and it was visualized using the online tool (https://www.chiplot.online/).

### 2.8 Transient expression of exogenous proteins in tobacco

Transient expression of *AsCOMT23* and *AsCOMT27* in tobacco leaves was achieved via Agrobacterium-mediated infiltration. Lignin content was subsequently measured to validate their roles in lignin biosynthesis. The method for transient expression of exogenous proteins in tobacco was adapted from the procedure described (Udawat et al., 2016).

### 2.9 Data analysis

Data performed using the Windows version of SPSS 24.0 (SPSS Inc., USA), with measurements presented as means ± standard errors. Statistical significance between control and treatment group measurements at different times was assessed using *t*-tests. Figures were created using GraphPad Prism 8.0.2 software.

## 3 Results

### 3.1 Identification and analysis of physicochemical properties of oat *COMT* genes

**Figure 1:**
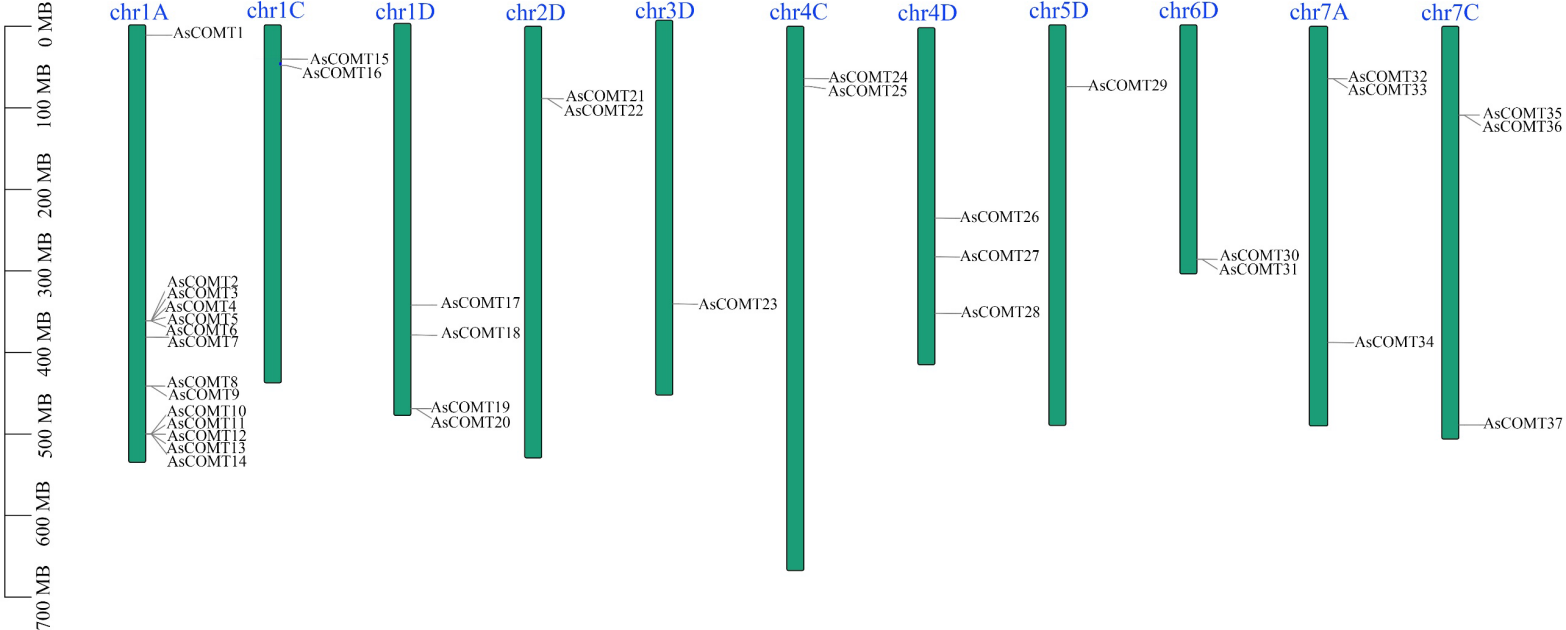
Distribution of *AsCOMTs* genes across oat chromosomes. Blue bars represent the nine chromosomes that contain *AsCOMT* genes. The scale indicates the length of each chromosome, and the black lines mark the position of each *AsCOMT* gene. The green bars in the figure represent the 11 chromosomes containing the AsCOMT gene, with the blue text above indicating the chromosome names; the black lines mark the position of each AsCOMT gene. The scale on the left side of the figure indicates the chromosome length in Mb, allowing for the inference of the relative positions and intervals between the genes.

Using comparative approaches, 37 *COMT* genes were identified in the oat genome, and their nomenclature was based on the random distribution of the *AsCOMT* genes across 11 chromosomes. These genes were named *AsCOMT1–37*. The *AsCOMT* genes were distributed across all 11 chromosomes (**Figure 1**). The proteins encoded by these genes ranged from 99 amino acids (AsCOMT8) to 420 amino acids (AsCOMT15), with an average length of approximately 351 amino acids. Their molecular weights ranged from 3.8 KDa to 44 KDa (**Supplementary Table S1**). Except for AsCOMT26, AsCOMT4, and AsCOMT36, which had theoretical isoelectric points (PIs) of less than 5, the predicted PIs of the other AsCOMT proteins were greater than 5, indicating they are acidic in nature. All AsCOMTs were predicted to lack transmembrane helices (TMHs) and localize in the cytoplasm or extracellular space.

### 3.2 Predictions of AsCOMT protein secondary and tertiary structures

The secondary structures of all 37 AsCOMT proteins were predicted to include α-helices, irregular curls, β-turns, and extended chains, though the proportions of each structure varied (**Supplementary Table S1**; **Supplementary Figure S2**). For all AsCOMT proteins, α-helices were the most prominent secondary. The proportions of irregular curls ranged from 28.44% to 38.38%, with an average of 31.70%. β-turn structures were the smallest contributors to the secondary structure in all AsCOMT proteins. The proportions of extended chains fluctuated between 9.54% to 20.20%. Tertiary structure predictions (**Supplementary Figure S1**) showed that the tertiary structures of AsCOMT4, AsCOMT5, AsCOMT6, and AsCOMT7 were highly similar, as were those of AsCOMT30 and AsCOMT31. The remaining proteins displayed significant differences in their tertiary structures, though α-helical structures were the dominant feature in all cases.

### 3.3 Conserved motif analysis of oat AsCOMT protein sequences

**Figure 2:**
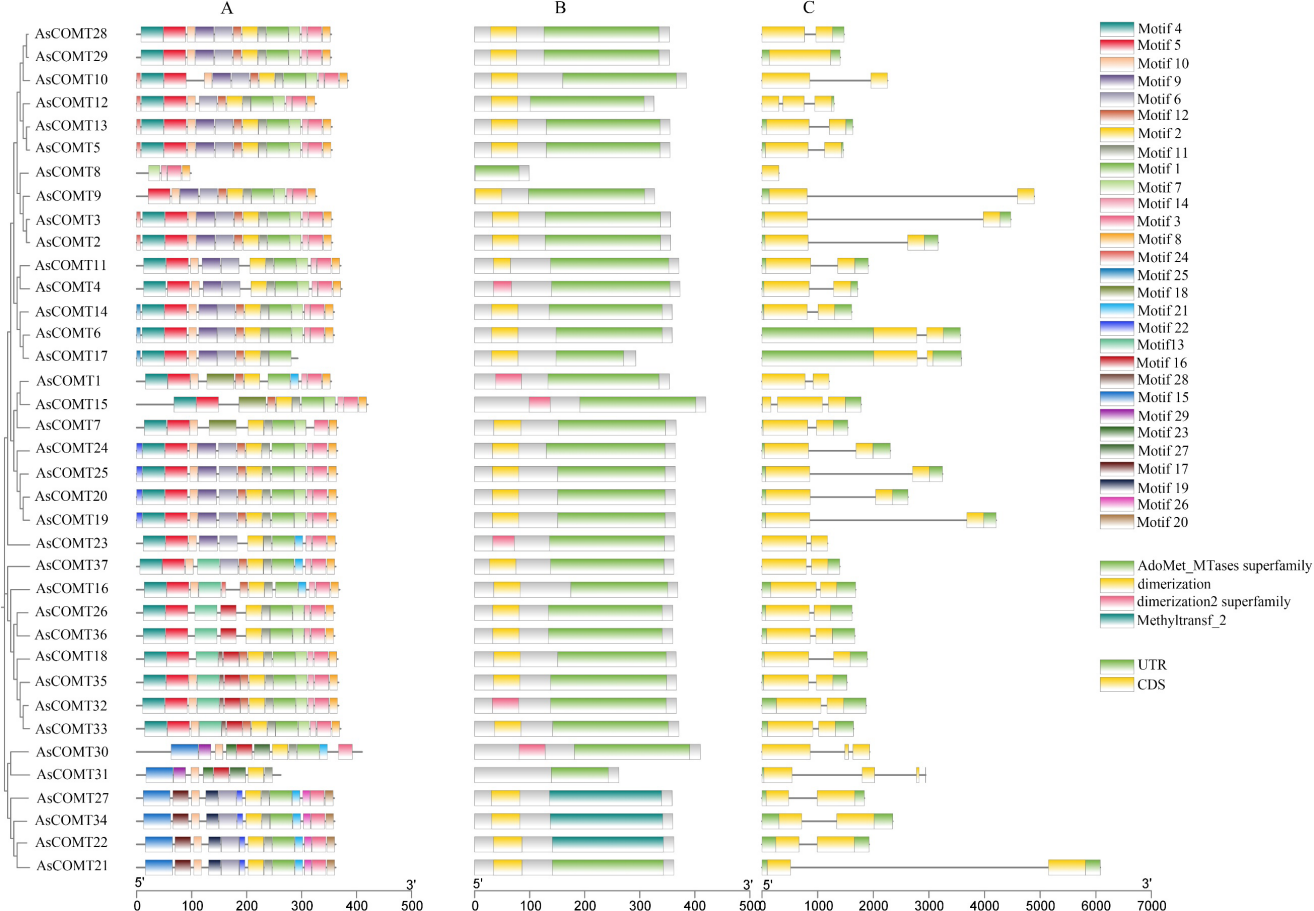
Conserved motif diagram of oat AsCOMT proteins. **(A)** Conserved motif analysis of AsCOMT family proteins; **(B)** Conservative structural domain analysis; **(C)** Gene structure analysis.

To further investigate the evolutionary diversity of the AsCOMT family, conserved motifs in AsCOMT proteins were analyzed using MEME online software, resulting in the identification of 29 distinct conserved motifs (labeled motif 1–29) (**Figure 2**). The lengths of these motifs ranged from 8 and 50 amino acids, with motif 15 being 50 amino acids long (**Additional file 1: Figure S3**). The number of conserved domains varied across different genes. Among all the motifs, motifs 1, 2, and 11 were the most frequently occurring, suggesting that they represent characteristic motifs on the AsCOMT family. In terms of genetic structure, members of the AsCOMT gene family all contained 1–3 coding sequences (CDS), with variation due to exon insertions or deletions and different subfamilies of AsCOMT during evolution. This functional diversity and subfunctionalization of proteins led to significant differences in amino acid length and composition. Except for AsCOMT29 and AsCOMT8, the remaining 35 genes contain introns. Additionally, except for five genes (AsCOMT1, 8, 10, 23, and 30), all other 32 AsCOMT genes contain untranslated regions (UTRs). Among these, six oat COMT proteins (AsCOMT32, 30, 23, 1, 4, and 15) contain a dimerization 2 superfamily conserved domain, three oat COMT proteins (AsCOMT22, 27, and 34) contain a Methyltransfer2 conserved domain, and all 34 COMT proteins, except AsCOMT22, 27, and 34, contain an AdoMet_MTases superfamily conserved domain. Furthermore, all 34 COMT proteins, except AsCOMT8, 30, and 31, contain a dimerization conserved domain.

### 3.4 Phylogenetic analysis of COMT protein sequences

**Figure 3:**
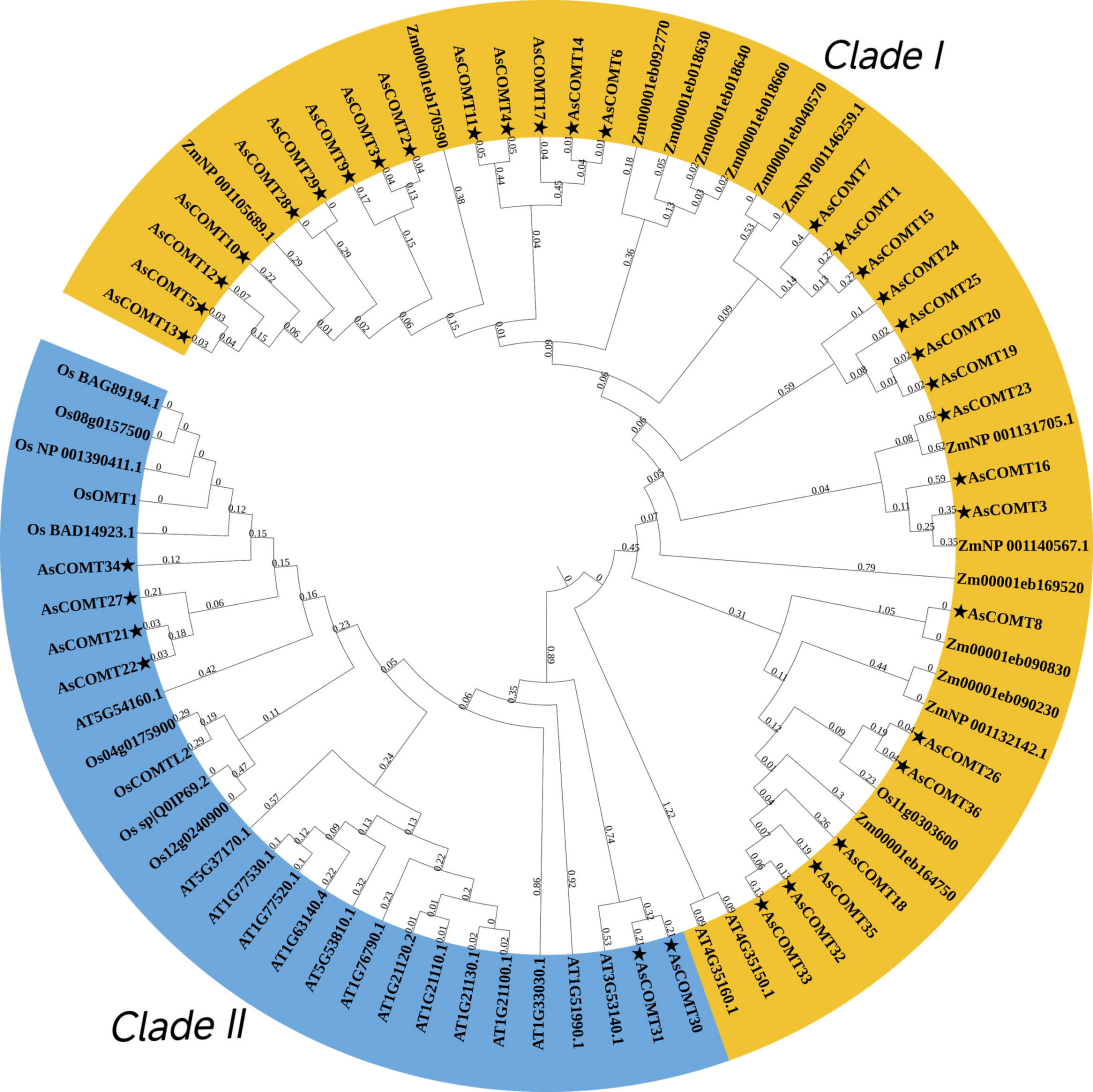
Phylogenetic analysis of COMT protein sequences in oat, *A. thaliana*, *O. sativa* and *Z. mays*. Oat AsCOMT proteins are marked with stars.

To better understand the similarities and differences in COMT between oats and other plants, a phylogenetic tree was constructed using 37 oat COMT proteins and 44 protein sequences from *A. thaliana* (16 sequences), *Z. mays* (18 sequences), and *O. sativa* (10 sequences) (**Figure 3**). The phylogenetic analysis of AsCOMT revealed that 37 oat COMT protein sequences can be divided into two groups: Clade I, which contain 31 AsCOMT proteins, and Clade II, which consists of the remaining 6 AsCOMT proteins. The oat COMT gene family is concentrated in these two groups, which are relatively conserved in the evolutionary process and exhibit a high degree of similarity. The analysis shows that oat COMTs are closely related to those of *Z. mays* and *O. sativa*, while *A. thaliana* COMTs cluster separately from both AsCOMTs, OsCOMTs, and ZmCOMTs. This suggests that oats share a more distant genetic relationship with *A. thaliana*, highlighting significant differences in the COMT genes between oats and *A. thaliana.*

### 3.5 Identification of cis-acting elements in AsCOMTs

**Figure 4:**
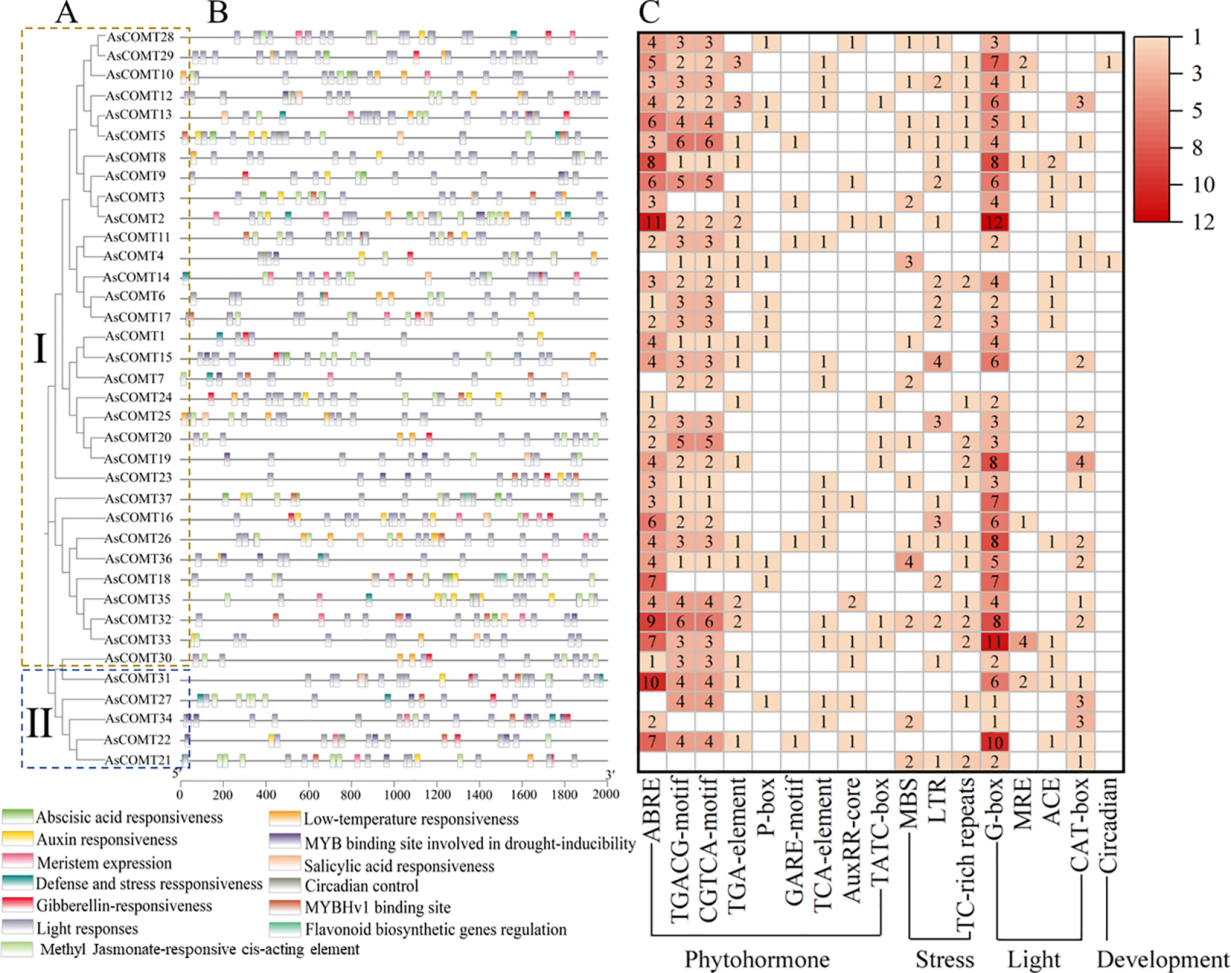
The cis-acting regulatory elements contained in the 2 kb promoter regions of the *AsCOMT* genes. **(A)** A phylogenetic tree was reconstructed using the full-length sequences of AsCOMT proteins, with different color backgrounds representing different groupings. **(B)** The distributions of cis-regulatory elements within the promoter regions of the *AsCOMT* genes, with different colored boxes representing different functional elements. **(C)** Statistics on the number of cis-acting elements of the *AsCOMT* genes. The numbers in the heat map box indicate the number of identified elements, while empty boxes indicate that no corresponding elements were identified.

The cis-acting elements identified in promoter regions of the *AsCOMT* genes were associated with light response, growth and development, hormone response, and abiotic stress response (**Figure 4C**), highlighting their potential as hubs in oat defense signaling. The analysis shows that, except for *AsCOMT21*, the remaining 36 *AsCOMT* genes contain 3 to 25 photo responsive elements. Additionally, 29 genes contained 1 to 3 auxin-responsive elements, suggesting involvement in growth-defense trade-offs. Twenty-one genes contained 1 to 2 gibberellin-responsive elements. Several genes also contained Methyl Jasmonate-responsive cis-acting element (MeJA-responsive elements), linking AsCOMT to stress-induced hormone crosstalk, salicylic acid responsive elements (SA-responsive elements), and abscisic acid responsive elements (**Figures 4B, C**). MBS (drought) and LTR (low temperature) elements were detected in 12 and 8 genes, respectively, indicating broad stress adaptability. Furthermore, *AsCOMT18* and *AsCOMT37* contained cis-acting elements related to flavonoid biosynthesis, while 17 genes contained 1 to 2 defense and stress response elements, directly linking AsCOMT to pathogen attack responses. Overall, the promoter regions of these *AsCOMT* genes were found to contain a variety of cis-acting regulatory elements, suggesting their potential involvement in multiple biological processes and regulatory pathways.

### 3.6 Synteny analysis of COMT family genes

**Figure 5:**
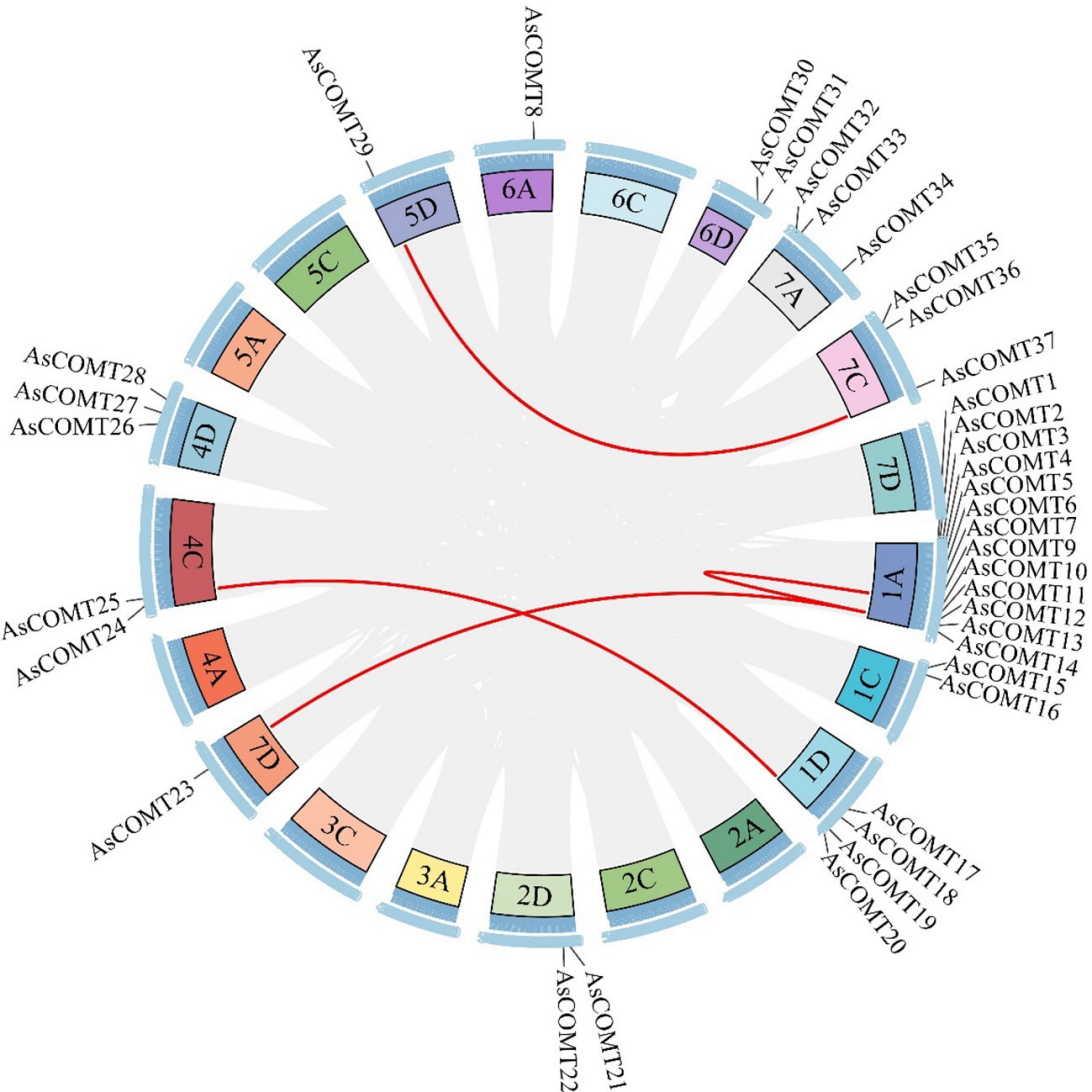
The synteny blocks of COMT genes in oat. Analysis of intrachromosomal fragment duplication of COMT genes in the oat genome. The grey lines represent all synteny blocks, while the red lines specially highlight the duplicated pairs among the 37 AsCOMT genes.

To explore the evolutionary relationships within AsCOMT family and between AsCOMTs and COMTs from other plant species, we analyzed the duplication events in the oat COMT gene family. The analysis revealed significant segment duplications among AsCOMT family members. Specifically, AsCOMT4 and AsCOMT11, AsCOMT11 and AsCOMT23, AsCOMT19 and AsCOMT25, and AsCOMT29 and AsCOMT37 all underwent duplication (**Figure 5**). Additionally, collinearity maps of the oat genome were constructed with two monocotyledons (*O. sativa* and *Z. mays*) and one dicotyledon (*A. thaliana*) (**Supplementary Figure S4**). The AsCOMT gene family exhibited strong collinearity with COMT genes from other monocotyledons, showing the strongest collinearity with *O. sativa* (8 pairs), followed by *Z. mays* (5 pairs). However, no collinearity was found between AsCOMT and the dicotyledonous plant *A. thaliana*. In summary, the oat COMT family members undergo significant segment duplications within the family and with other species, suggesting that some family members are homologous to segments from other species.

### 3.7 Prediction of interaction networks of AsCOMT proteins

**Figure 6:**
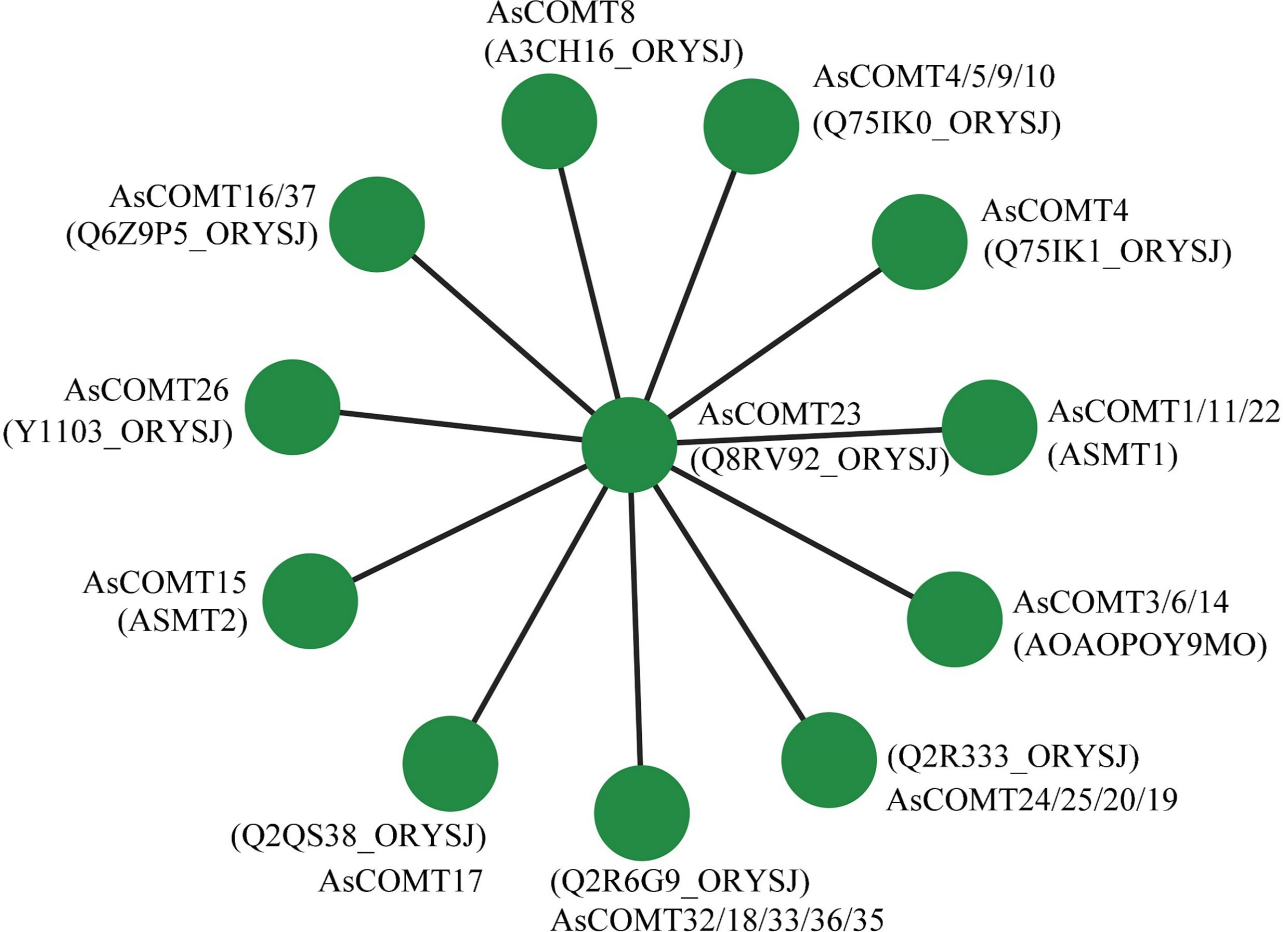
The predicted interaction network of AsCOMT proteins based on interactions of their orthologs in *O. sativa (Japonica)*.

Protein-protein interaction networks of AsCOMTs were predicted using the STRING database (confidence score >0.7), based on orthologous relationships with *O sativa* COMT proteins (**Figure 6**). The network revealed a central hub composed of AsCOMT23 and six core interactors (AsCOMT4/5/9/10/16/37), all sharing high sequence similarity and clustering within Clade I. AsCOMT23 as a central node suggests AsCOMT23 may act as a scaffold for multi-enzyme complexes during lignin monomer methylation.

### 3.8 Organizational expression analysis of AsCOMTs

A total of 37 AsCOMTs were retrieved from the transcriptome database, with their expression levels in different tissues represented in FPKM (Fragments Per Kilobase of Exon Model per Million Mapped Fragments). A heatmap was constructed based on the expression data of these genes across various tissues (**Supplementary Figure S5**). The analysis revealed that some *AsCOMT* genes are expressed at low levels across various tissues, suggesting they may be induced to express under specific conditions. Certain genes demonstrated strong tissue specificity: for instance, *AsCOMT3*, *AsCOMT37*, *AsCOMT17*, *AsCOMT14*, and *AsCOMT16* exhibited higher expression levels in the crown; *AsCOMT21*, *AsCOMT23*, *AsCOMT22*, *AsCOMT2*, and *AsCOMT1* had elevated expression in the roots; and *AsCOMT23* was notably expressed in the leaf. Additionally, *AsCOMT37* showed high expression levels across multiple tissues, highlighting its crucial role in oat growth and development.

#### 3.8.1 Response to powdery mildew

**Figure 7:**
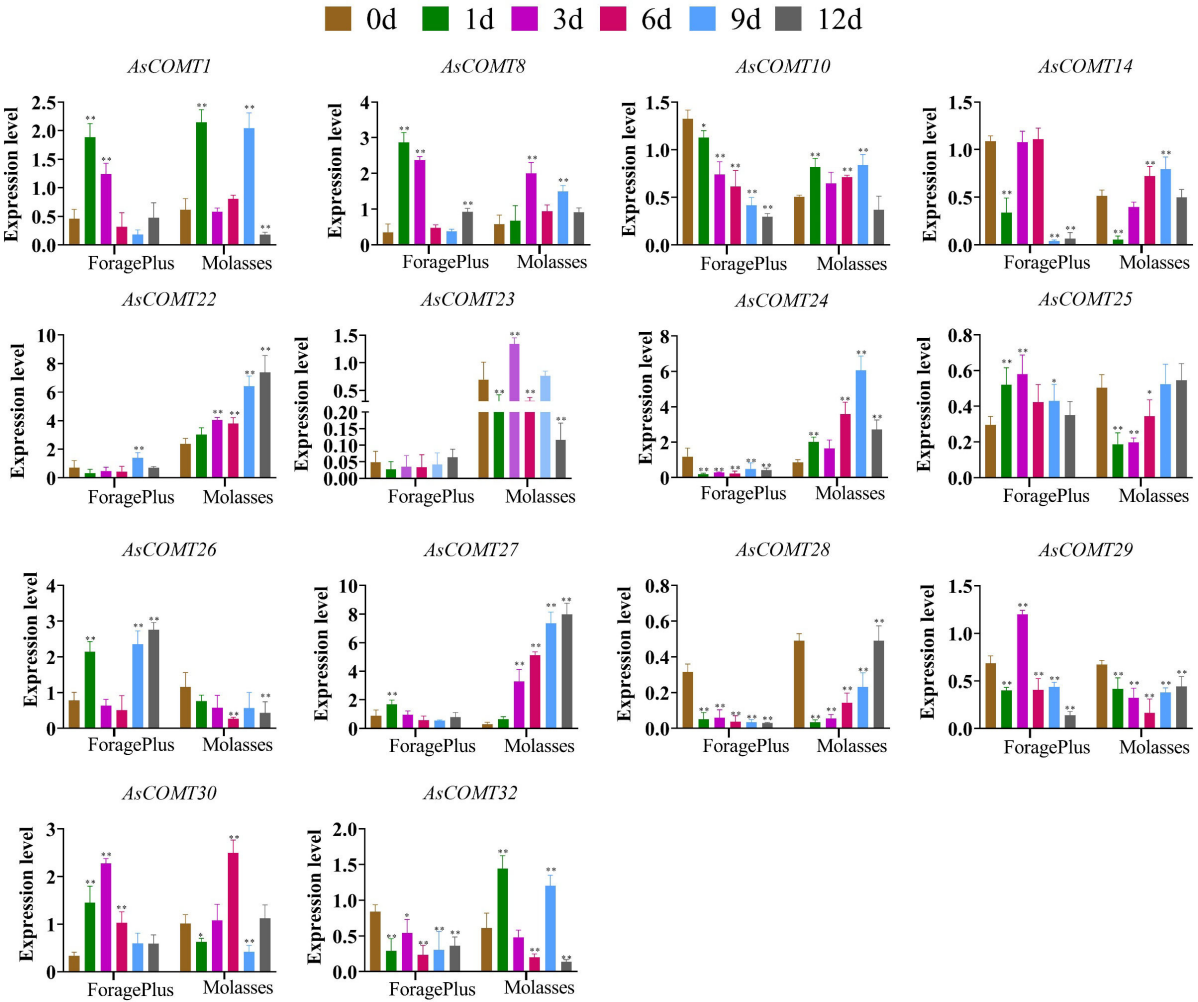
Expression of *AsCOMT* genes in two oat cultivars, ‘ForagePlus’ and ‘Molasses’, following infection with Powdery mildew. Significant differences are indicated by asterisks (*p < 0.05, **p < 0.01).

The expression of *AsCOMT* genes was assayed in two oat cultivars, ‘ForagePlus’ and ‘Molasses’, following inoculation with powdery mildew. Several *AsCOMT* genes showed significant changes in expression levels after infection (**Figure 7**). In ‘ForagePlus’, the expression of *AsCOMT8*, *25*, and *30* was significantly up regulated in the leaves. In contrast, in ‘Molasses’, the expression of *AsCOMT22*, and *27* was significantly up regulated, high expression in disease - resistant varieties may be related to enhanced disease resistance. Additionally, *AsCOMT28*, and *10* were significantly down-regulated in ‘ForagePlus’ leaves but showed significant up-regulation in ‘Molasses’ leaves. These results indicate cultivar-specific differences in the regulation of AsCOMT genes following infection. After the ‘ForagePlus’ variety is infected with powdery mildew, the expression of *AsCOMT8* begins to show an upward trend on the 1st day. This is likely an early response of the plant to the invasion of pathogenic bacteria, initiating relevant defense mechanisms. On the 3rd day, the expressions of *AsCOMT25* and *AsCOMT30* are significantly upregulated. At this time, it may be that the defense response is further strengthened, and more defense-related genes are activated. After 6 days, the expressions of some genes start to fluctuate. For example, the expression of *AsCOMT24* is downregulated, which may be the plant’s way of regulating the intensity of the defense response to avoid excessive energy consumption. On the 9th and 12th days, the expressions of some genes gradually recover or remain at a certain level, reflecting the dynamic regulation of the plant during the process of adapting to the infection of pathogenic bacteria.

#### 3.8.2 Response to leaf spot stress disease

**Figure 8:**
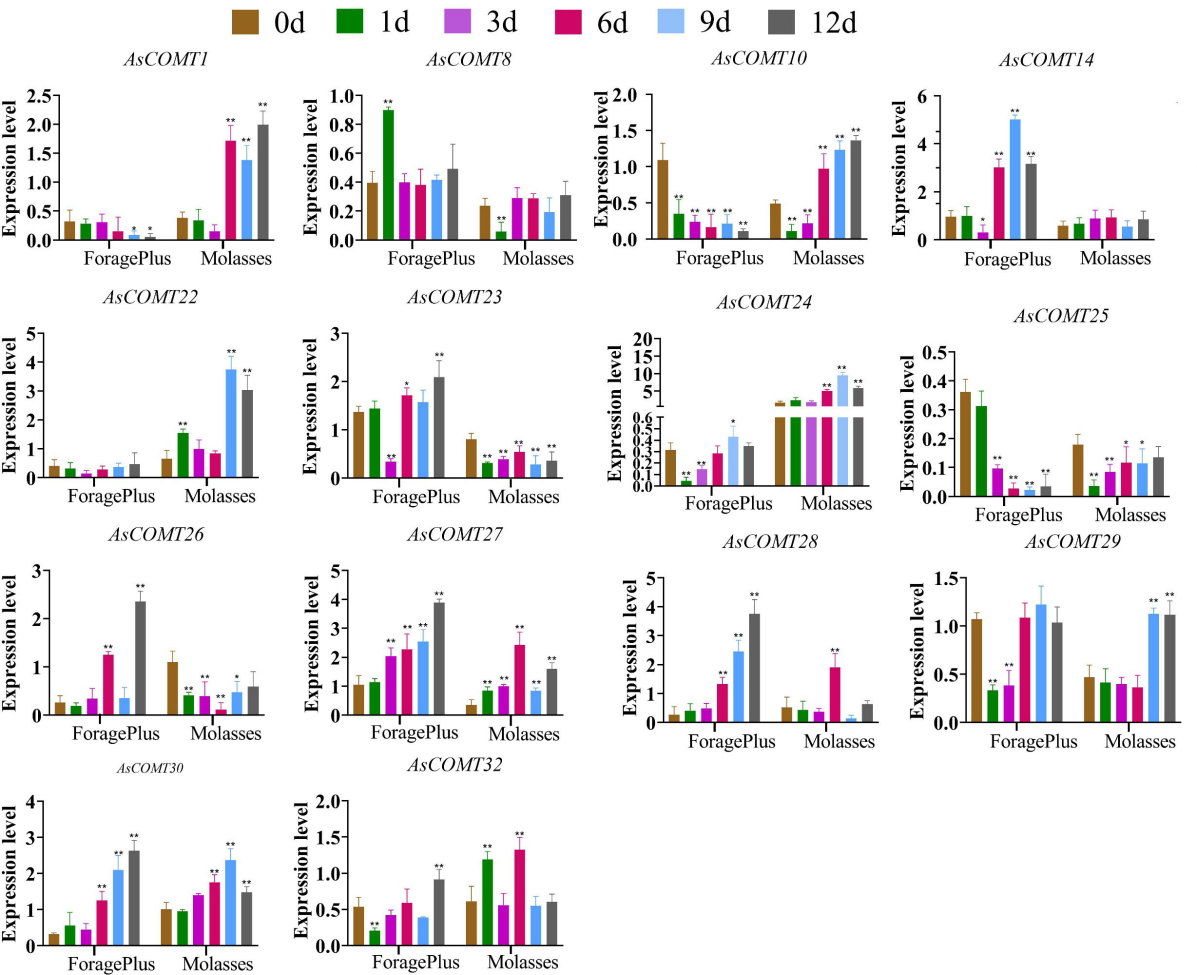
Expression of AsCOMT genes in two oat cultivars, ‘ForagePlus’ and ‘Molasses’, following infection with leaf spot. Significant differences are indicated by asterisks (*p < 0.05, **p < 0.01).

Six days after inoculation, the relative expression levels of *AsCOMT30*, *28*, and *14* in ‘ForagePlus’ were significantly higher compared to the control (*p* < 0.05) (**Figure 8**). Similarly, the relative expression levels of *AsCOMT30*, and *10* in ‘Molasses’ were significantly increased as compared to the control (*p* < 0.05). *AsCOMT30* and *27* were significantly up regulated in both cultivars. The expression of *AsCOMT22* remained stable in ‘ForagePlus’ after infection, while it was significantly up regulated in the leaves of ‘Molasses’.

When infected with powdery mildew, *AsCOMT24* expression is significantly upregulated in disease-resistant varieties but significantly downregulated in susceptible varieties. By contrast, when infected with leaf spot disease, *AsCOMT24* expression is significantly upregulated in both varieties. This differential expression pattern indicates that *AsCOMT24* exhibits variety-specific responses to different diseases and may play different roles in different disease resistance mechanisms.

#### 3.8.3 Changes in lignin content

**Figure 9:**
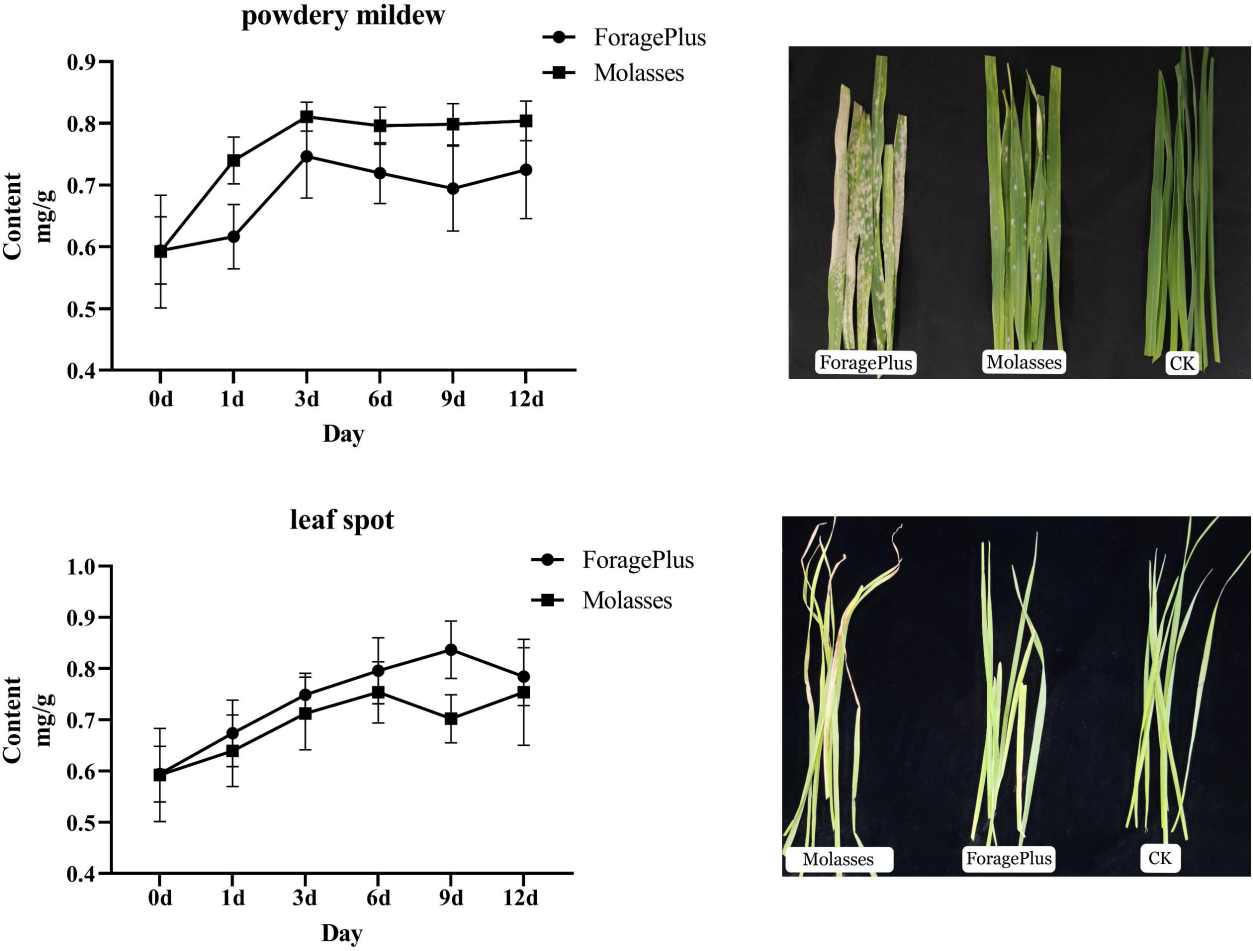
Lignin content dynamics in oat leaves under powdery mildew and leaf spot stress.

When the oat cultivars ‘ForagePlus’ and ‘Molasses’ were not infected, there was little difference in lignin content between their leaves (**Figure 9**). However, after infection with the pathogen, the lignin content increased. Specifically, after infection with powdery mildew, the lignin content in ‘ForagePlus’ increased initially, peaked around the third day, then decreased, and rose again after 9 days. In contrast, ‘Molasses’ exhibited a steady increase in lignin content after the third day, which stabilized thereafter. At all time points, the lignin content in ‘Molasses’ was higher than in ‘ForagePlus’, indicating that ‘ForagePlus’ is more susceptible to powdery mildew.

**Figure 10:**
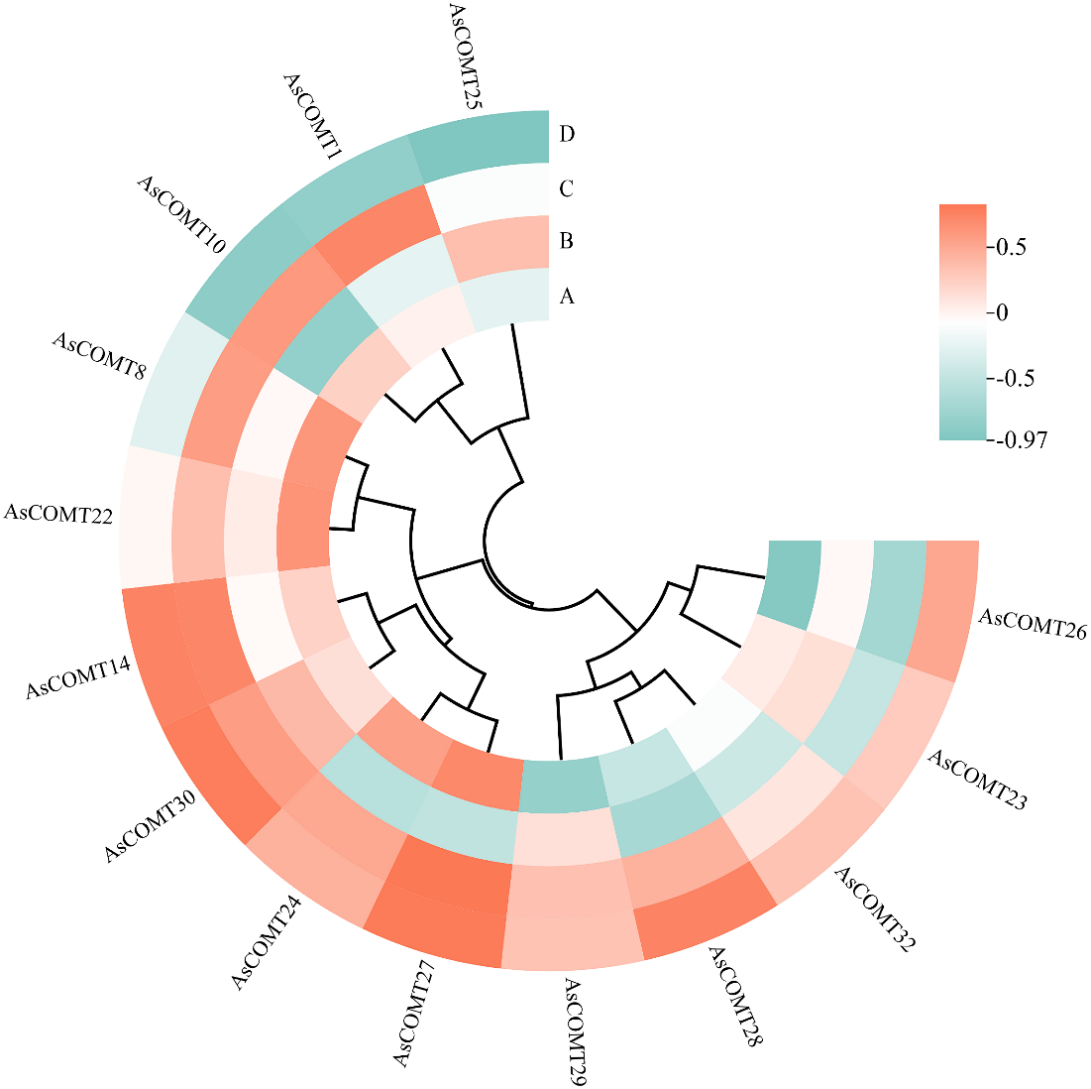
Correlation analysis between the lignin content and the expression of AsCOMT genes. **(A)** The oat variety ‘Molasses’ infected with powdery mildew, **(B)** the oat variety ‘ForagePlus’ infected with powdery mildew, **(C)** the oat variety ‘Molasses’ infected with leaf spot, **(D)** the oat variety ‘ForagePlus’ infected with leaf spot.

After infection with leaf spot disease, the lignin content of ‘ForagePlus’ increased, peaking at 9 days before decreasing, while ‘Molasses’ saw an increase in lignin content that started to decrease at 6 days and then rose again after 9 days. At every time point, the lignin content in ‘ForagePlus’ was higher than in ‘Molasses’, indicating that ‘ForagePlus’ is more susceptible to powdery mildew, but ‘Molasses’ exhibited a stronger resistance to leaf spot. After the ‘Molasses’ variety is infected with leaf spot disease, the lignin content increase slightly on the 1st day. This is likely the initial manifestation of the plant starting lignin synthesis after perceiving the pathogen. The upward trend of the lignin content accelerates on the 3rd day. This is probably because the expressions of related genes are upregulated at this time, promoting the synthesis of lignin. The lignin content reaches a small peak on the 6th day and then decreases somewhat. This may be due to the plant balancing lignin synthesis with other physiological processes. The lignin content starts to rise again on the 9th day, probably to further enhance the defensive ability of the cell wall in response to the continuous infection of the pathogen. Overall, lignin content increases after infection, and the resistant variety consistently showed higher lignin content in response to both diseases compared to the susceptible variety. This suggests that lignin may play a role in the response of oats to powdery mildew and leaf spot disease. Furthermore, correlation analysis between lignin content and AsCOMT gene expression revealed a positive correlation with AsCOMT30 in both the susceptible and resistant varieties. In contrast, genes such as *AsCOMT14*, *AsCOMT24*, and *AsCOMT27* exhibited a stronger positive correlation in the resistant variety than in the susceptible one (**Figure 10**). These genes may catalyze key reactions in the lignin synthesis pathway, increasing the synthesis of lignin monomers. Eventually, this leads to an increase in lignin content, enhancing the resistance of oats to diseases.

#### 3.8.4 Instantaneous expression of lignin content in tobacco with *AsCOMT23* and *AsCOMT27*

**Figure 11:**
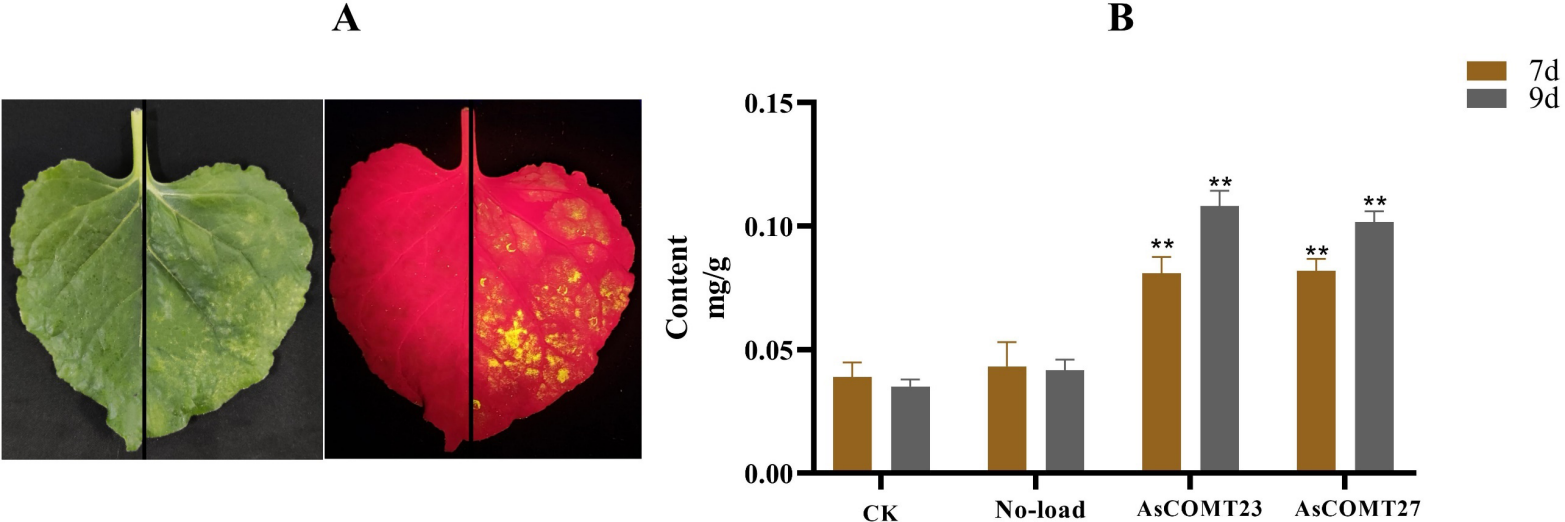
Tobacco leaves transiently expressing *AsCOMT23* and *AsCOMT27* **(A)** and its lignin content **(B)**. **(A)** The left image shows the state of tobacco leaves after infection under normal light conditions, while the right image shows the state of tobacco leaves after infection under fluorescent light. The diagonal line indicates that the upper part was not injected with Agrobacterium, while the lower part was injected with Agrobacterium; **(B)** Changes in lignin content in tobacco leaves after 7 and 9 days of transient expression (**p < 0.01).

Due to the significant increase in expression levels of *AsCOMT23* in resistant varieties and the significant decrease in susceptible varieties after leaf spot disease infection, and the significant increase in expression levels of *AsCOMT27* in resistant varieties and the maintenance of expression levels in susceptible varieties after powdery mildew infection, these two genes were selected for overexpression in tobacco. After injecting the tobacco epidermis, the infection was observed using fluorescence, resulting the transient expression of *AsCOMT23* and *AsCOMT27* in tobacco (**Figure 11A**). After infecting tobacco with the empty vector Pcan for 7 and 9 days, the lignin content in *AsCOMT23-*and *AsCOMT27-*expressing tobacco showed no significant changes compared to the uninfected controls. However, after infecting tobacco with the expression vectors Pcan-AsCOMT23 and Pcan-AsCOMT27 for 7 and 9 days, the lignin content in tobacco with transient expression of *AsCOMT23* and *AsCOMT27* showed varying degrees of change (**Figure 11B**). Specifically, at 7 days, the lignin content in *AsCOMT23*-expressing tobacco increased by 51.82% compared to the uninfected control and 46.64% compared to the empty vector-infected control, respectively. In *AsCOMT27*-expressing tobacco, lignin content increased by 52.39% compared to the uninfected control and 47.27% compared to the empty vector-infected control, respectively, while in *AsCOMT27*-expressing tobacco, the lignin content increased by 65.49% and 58.98%, respectively.

## 4 Discussion

In the landscape of COMT gene family research, existing studies have predominantly focused on model plants like *A. thaliana*, *O. sativa*, and *Z. mays*. These studies have provided valuable insights into the basic functions and regulatory mechanisms of the COMT gene family in general plant processes. However, to our knowledge, this study represents the first systematic exploration of the COMT gene family in oat, with a unique focus on its role in fungal resistance. In this study, we performed a comprehensive genome-wide analysis of the COMT gene family in common oat and investigated its potential role in responding to fungal infections, specifically powdery mildew and leaf spot. Our results provide valuable insights into the structure, evolutionary relationships, and functional dynamics of the COMT gene family in oats, offering a foundation for future research into their involvement in disease resistance. A total of 37 oat AsCOMT family proteins were identified in oat, a number that is comparable to or slightly higher than those found in other cereal crops such as *O. sativa* (33) (Liang et al., 2022) and *Z. mays* (18) (Yang et al., 2017). The chromosomal distribution of these genes suggests a random dispersal across the oat genome, which reflects the complexity of gene duplication events during oat genome evolution (Kamal et al., 2022). Phylogenetic analysis classified the AsCOMT genes into two major clades, consistent with findings in other plant species (Xu et al., 2023), indicating the evolutionary conservation of COMT functions. The classification of these genes into two subgroups suggests functional specialization, likely driven by gene duplication and divergence over evolutionary time. The close phylogenetic relationship of oat COMT genes with those from *Z. mays* and *O. sativa* highlights the shared ancestry of this gene family across monocots and dicots, suggesting that the roles of COMT in lignin biosynthesis and plant defense may be conserved. This conservation is likely essential for maintaining key metabolic pathways such as lignin formation, which is vital for structural integrity and pathogen resistance in plants (Ninkuu et al., 2022).

The expression analysis of *AsCOMT* genes in response to powdery mildew and leaf spot infections revealed that several genes, including *AsCOMT22, AsCOMT24, AsCOMT27, AsCOMT28*, and *AsCOMT30*, were significantly upregulated in resistant oat varieties after pathogen infection (**Supplementary Figure S5**). This suggests that these COMT genes play a crucial role in strengthening plant defenses, likely by promoting lignin biosynthesis, which reinforces the cell walls against pathogen invasion. In a related study, silencing *ZmPAL6*, *ZmCOMT*, and *ZmCCoAOMT2* genes in *Z. mays* using virus-induced gene silencing (with *ZmCCoAOMT2* being functionally similar to ZmCOMT and acting downstream in the lignin biosynthesis pathway) (Cao et al., 2023; Parvathi et al., 2001) increased the susceptibility of the plants to *Fusarium verticillioides* infection. This led to more severe root rot symptoms in the silenced plants compared to controls. These genes (*ZmPAL6*, *ZmCOMT*, and *ZmCCoAOMT2*) contribute to the defense response by regulating lignin deposition in root cell walls after fungal invasion. Notably, these genes function in different cell types, as the knockout of *ZmCOMT* caused a sharp decrease in lignin content and staining in the periderm cells, vascular bundles, and endodermis, affecting 3 to 4 cell layers. These findings support the idea that genes involved in the phenylpropanoid pathway, such as *ZmPAL6*, *ZmCOMT*, and *ZmCCoAOMT2*, regulate lignin biosynthesis in different cell types and play a critical role in disease defense responses. In *Z. mays*, (encoding caffeoyl-CoA O-methyltransferase) confers quantitative resistance to both southern leaf blight and gray leaf spot, with lignin levels positively correlated to disease resistance (Osakabe et al., 1999). This suggests that, similar to *Z. mays*, the upregulation of *AsCOMT* genes in oats during fungal infection may enhance lignin production and contribute to disease resistance.

Previous studies in other crops have shown that lignin content increases in response to biotic stress (Han et al., 2022; Li et al., 2017), and that COMT genes play a key role in this process by catalyzing the methylation of intermediates in the lignin biosynthetic pathway (Yang et al., 2017). Our findings are consistent with these observations. We also found that after infecting tobacco with the expression vectors Pcan-AsCOMT23 and Pcan-AsCOMT27 for 7 and 9 days, the lignin content in tobacco leaves with transient expression of *AsCOMT23* and *AsCOMT27* significantly increased. Huang et al. (2024) indicated through bioinformatics analysis that LcCOMT can participate in the biosynthesis of melatonin and lignin through O-methylation, and has a high preference for the lignin biosynthesis precursor, caffeic acid. In this study, we found that overexpressing *AsCOMT23* and *AsCOMT27* significantly increased the lignin content in tobacco leaves. This enhanced lignin production would likely help limit pathogen penetration and spread, thereby improving resistance. This is similar to Huang et al. (2024), indicating that the COMT gene in different plants may participate in the pathogen resistance process by affecting lignin synthesis. However, there are also differences. Huang et al. (2024) primarily found that LcCOMT in Ligusticum chuanxiong has a high preference for the lignin biosynthesis precursor caffeic acid, thereby establishing a link between LcCOMT and lignin biosynthesis. In contrast, this study directly verified the role of the AsCOMT gene in lignin synthesis through pathogen inoculation experiments, providing more direct evidence for the function of COMT genes in plant disease resistance mechanisms. Yang et al. (1986) studied wheat powdery mildew and found that different wheat varieties showed varying increases in lignin content after inoculation, and these increases were positively correlated with the disease resistance of the wheat varieties. Promoter analysis of the *AsCOMT* genes revealed numerous cis-regulatory elements related to plant hormone signaling (e.g., abscisic acid, SA-responsive elements) and stress responses. These elements are likely involved in regulating gene expression in response to biotic stresses, such as fungal infections. The differential expression patterns observed in resistant and susceptible oat varieties suggest that these regulatory elements may play a critical role in modulating the intensity and timing of the defense response. The involvement of SA-responsive elements and Jasmonic acid signaling pathways in mediating plant defense mechanisms has been well-documented (Mur et al., 2013; Yuan et al., 2019; Kachroo and Kachroo, 2012). The presence of SA- and Jasmonic acid responsive elements in the promoters of AsCOMT genes suggests that these hormone-mediated defense pathways could be responsible for activating COMT gene expression during pathogen attack. This discovery provides a new perspective on how *AsCOMT* genes are regulated during fungal infections in oats, which has not been explored in previous COMT family studies, especially in the context of oat-specific responses to biotic stress. Further studies are needed to explore the specific hormonal regulation of COMT genes in oats to better understand their roles in defense signaling networks. Understanding these mechanisms could provide valuable insights into improving disease resistance in oats and other crops.

Currently, there have been no reported studies on QTLs or Multivariate Trait Association Study (MTAS) related to the COMT gene in oats. However, given the important role of the COMT gene in plant lignin synthesis and disease resistance, conducting such research is of significant importance. In future research, although the AsCOMT gene family has been identified through bioinformatics analyses, constructing different oat populations (e.g., recombinant inbred lines, doubled haploid populations) and integrating high-density molecular marker maps with phenotypic data will be crucial for mapping QTLs that may include COMT genes and clarifying their genetic contributions to traits like disease resistance or lignin content. Additionally, MTAS in oat germplasm can help identify molecular markers linked to COMT expression or function, which would support marker-assisted breeding. While no COMT-associated QTLs have been reported to date, this approach will establish a foundation for validating the role of COMT genes in genetic regulatory networks. Additionally, based on the analysis results of the AsCOMT gene family in this study, future research could focus on the stability of COMT gene-related QTLs and MTAS under different environmental conditions, to better understand the genetic regulatory mechanisms of the COMT gene in oat growth and development and in response to biotic stress.

This study fills a significant gap in the understanding of the COMT gene family by focusing on oats and establishing links between gene evolution, expression dynamics, and cis-regulatory elements in the context of fungal resistance. The identification of *COMT* genes associated with disease resistance in oats provides valuable genetic resources for oat breeding programs. Given the crucial role of lignin in plant defense, AsCOMT genes (particularly those highly responsive to fungal infection) could serve as potential targets for developing disease-resistant oat varieties through marker-assisted selection or genetic engineering. In this study, genetic engineering techniques were employed via transient overexpression of AsCOMT23 and AsCOMT27 in tobacco leaves. The differential expression of *COMT* genes between resistant and susceptible oat varieties highlights the potential to use these genes as molecular markers for screening oat germplasm with enhanced disease resistance. This approach could significantly accelerate the breeding process by enabling the early identification of resistant lines, thereby improving the efficiency of breeding programs focused on combating powdery mildew and leaf spot infections.

## 5 Conclusion

This study identified a total of 37 *AsCOMT* genes in oats. Subcellular localization predictions suggest that AsCOMTs proteins are located on the plasma membrane. Phylogenetic tree analysis divides the *AsCOMT* genes into two subfamilies, with high homology and strong conservation with the COMT protein families in maize and rice. The promoter regions of the *AsCOMT* genes contain cis-acting elements related to light response, growth and development, and stress responses. These findings suggest that *AsCOMT* genes play an active role in the oat plant’s response to biotic stress. Notably, *AsCOMT14, 22, 24*, and *27* exhibit significant differential expression under powdery mildew and leaf spot disease stress, making them important candidates for further study of oat responses to these biotic stresses. Moreover, the lignin content in disease-resistant oat varieties is higher than that in susceptible varieties. Overexpression of *AsCOMT23* and *AsCOMT27* in tobacco leaves resulted in a significant increase in lignin content. These results not only establish a link between COMT-mediated lignin synthesis and fungal resistance in oats but also provide potential genetic targets for molecular breeding of disease-resistant crops. Future studies focusing on the regulatory networks of these genes and their deployment in oat germplasm improvement will further solidify their practical applications in sustainable agriculture.

## Data Availability

The datasets presented in this study can be found in online repositories. The names of the repository/repositories and accession number(s) can be found in the article/**Supplementary Material**.
