# Supplementary material for: Genome-wide analysis of the COMT gene family in Avena sativa: insights into lignin biosynthesis and disease defense mechanisms
Source: Front Plant Sci. 2025 Jun 19;16:1609698. doi: 10.3389/fpls.2025.1609698 (PMC12222199; doi:10.3389/fpls.2025.1609698)
Supplement: Supplementary file 1 [file DataSheet1.docx]

Table S1 Physicochemical properties of AsCOMT proteins in oats.

| Gene Name | Number of amino acids | Molecular mass | Theoretical（PI） | Instability index | Fat index | Hydrophilicity | Transmembrane  helix | Subcellular localization |
| --- | --- | --- | --- | --- | --- | --- | --- | --- |
| AsCOMT1 | 365 | 39.58864 | 5.3 | 36.1 | 94.85 | 0.098 | 0 | Cytoplasmic |
| AsCOMT2 | 367 | 40.64724 | 5.81 | 37.97 | 101.69 | 0.06 | 0 | Cytoplasmic |
| AsCOMT3 | 410 | 44.05921 | 5.83 | 46.67 | 84.1 | -0.061 | 0 | Extracellular |
| AsCOMT4 | 354 | 38.86989 | 5.51 | 25.71 | 105.25 | 0.21 | 0 | Cytoplasmic |
| AsCOMT5 | 368 | 39.74554 | 5.22 | 24.73 | 88.1 | 0.092 | 0 | Cytoplasmic |
| AsCOMT6 | 359 | 39.87819 | 5.07 | 31.94 | 96.69 | 0.145 | 0 | Cytoplasmic |
| AsCOMT7 | 363 | 38.6934 | 5.31 | 42.63 | 95.18 | 0.166 | 0 | Cytoplasmic |
| AsCOMT8 | 99 | 11.26602 | 5.65 | 34.45 | 93.43 | -0.188 | 0 | Cytoplasmic |
| AsCOMT9 | 354 | 38.71769 | 5.61 | 35.44 | 96.41 | 0.132 | 0 | Cytoplasmic |
| AsCOMT10 | 355 | 38.64264 | 5.71 | 34.79 | 96.14 | 0.15 | 0 | Cytoplasmic |
| AsCOMT11 | 362 | 39296.46 | 5.55 | 45.18 | 90.55 | 0.084 | 0 | Extracellular |
| AsCOMT12 | 363 | 38443.16 | 5.21 | 38.7 | 95.98 | 0.228 | 0 | Cytoplasmic |
| AsCOMT13 | 360 | 38717.47 | 4.85 | 31.65 | 101.86 | 0.224 | 0 | Cytoplasmic |
| AsCOMT14 | 359 | 39240.43 | 5.22 | 37.88 | 90.19 | 0.122 | 0 | Extracellular |
| AsCOMT15 | 355 | 38290.16 | 5.35 | 37.77 | 107.15 | 0.246 | 0 | Cytoplasmic |
| AsCOMT16 | 326 | 35475.91 | 5.24 | 26.78 | 101.13 | 0.162 | 0 | Cytoplasmic |
| AsCOMT17 | 371 | 40692.63 | 5.05 | 45.27 | 93.83 | 0.025 | 0 | Cytoplasmic |
| AsCOMT18 | 327 | 35746.42 | 5.99 | 31.01 | 100.76 | 0.146 | 0 | Cytoplasmic |
| AsCOMT19 | 366 | 39516.23 | 5.09 | 39.68 | 94.43 | 0.176 | 0 | Cytoplasmic |
| AsCOMT20 | 359 | 39934.17 | 5.07 | 31.99 | 96.69 | 0.146 | 0 | Cytoplasmic |
| AsCOMT21 | 355 | 38386.34 | 5.2 | 35.24 | 106.03 | 0.226 | 0 | Cytoplasmic |
| AsCOMT22 | 373 | 40752.73 | 4.86 | 42.49 | 91.77 | 0.028 | 0 | Cytoplasmic |
| AsCOMT23 | 356 | 39023.4 | 5.72 | 37.23 | 103.57 | 0.156 | 0 | Cytoplasmic |
| AsCOMT24 | 356 | 38884.05 | 5.49 | 31.81 | 98.9 | 0.134 | 0 | Cytoplasmic |
| AsCOMT25 | 369 | 39268.22 | 5.37 | 45.61 | 96.78 | 0.203 | 0 | Cytoplasmic |
| AsCOMT26 | 420 | 45933.84 | 6.57 | 43.87 | 88.24 | 0.032 | 0 | Cytoplasmic |
| AsCOMT27 | 365 | 39486.61 | 5.29 | 31.26 | 97.78 | 0.155 | 0 | Cytoplasmic |
| AsCOMT28 | 365 | 39331.41 | 5.29 | 30.86 | 99.12 | 0.175 | 0 | Cytoplasmic |
| AsCOMT29 | 366 | 39586.82 | 5.72 | 36.53 | 105.85 | 0.189 | 0 | Cytoplasmic |
| AsCOMT30 | 293 | 32530.18 | 5 | 38.07 | 86.25 | -0.001 | 0 | Cytoplasmic |
| AsCOMT31 | 371 | 40830.37 | 5.27 | 39.36 | 100.4 | 0.109 | 0 | Cytoplasmic |
| AsCOMT32 | 360 | 38804.77 | 5.82 | 37.03 | 90.03 | 0.068 | 0 | Cytoplasmic |
| AsCOMT33 | 362 | 39141.99 | 5.38 | 30.11 | 92.27 | 0.143 | 0 | Cytoplasmic |
| AsCOMT34 | 360 | 38745.54 | 4.79 | 31.99 | 100.5 | 0.203 | 0 | Cytoplasmic |
| AsCOMT35 | 367 | 39668.79 | 5.79 | 32.3 | 100.68 | 0.109 | 0 | Cytoplasmic |
| AsCOMT36 | 362 | 39343.38 | 5.31 | 43.19 | 91.63 | 0.091 | 0 | Cytoplasmic |
| AsCOMT37 | 262 | 27475.57 | 5.1 | 48.82 | 92.82 | 0.206 | 0 | Cytoplasmic |

Table S2 Predictions of AsCOMT protein secondary structure.

| Gene name | α-helical structure (%) | Extension chain (%) | β-turn structure (%) | Random curl knot (%) |
| --- | --- | --- | --- | --- |
| AsCOMT1 | 49.32% | 13.42% | 7.40% | 29.86% |
| AsCOMT2 | 48.23% | 13.35% | 5.72% | 32.70% |
| AsCOMT3 | 44.39% | 13.17% | 6.10% | 36.34% |
| AsCOMT4 | 49.15% | 13.56% | 8.19% | 29.10% |
| AsCOMT5 | 49.15% | 13.56% | 8.19% | 29.10% |
| AsCOMT6 | 48.19% | 13.65% | 6.41% | 31.75% |
| AsCOMT7 | 48.48% | 13.50% | 6.61% | 31.40% |
| AsCOMT8 | 34.34% | 20.20% | 7.07% | 38.38% |
| AsCOMT9 | 48.31% | 13.84% | 5.93% | 31.92% |
| AsCOMT10 | 47.67% | 14.25% | 6.58% | 31.51% |
| AsCOMT11 | 48.34% | 14.92% | 6.63% | 30.11% |
| AsCOMT12 | 45.19% | 13.51% | 6.23% | 35.06% |
| AsCOMT13 | 48.89% | 12.78% | 6.11% | 32.22% |
| AsCOMT14 | 47.91% | 14.76% | 7.24% | 30.08% |
| AsCOMT15 | 48.45% | 14.08% | 7.32% | 30.14% |
| AsCOMT16 | 50.00% | 13.50% | 5.52% | 30.98% |
| AsCOMT17 | 46.09% | 14.29% | 7.01% | 32.61% |
| AsCOMT18 | 47.09% | 16.21% | 8.26% | 28.44% |
| AsCOMT19 | 48.91% | 13.11% | 6.01% | 31.97% |
| AsCOMT20 | 50.42% | 12.81% | 6.69% | 30.08% |
| AsCOMT21 | 50.14% | 13.24% | 7.61% | 29.01% |
| AsCOMT22 | 48.26% | 14.21% | 6.70% | 30.83% |
| AsCOMT23 | 49.44% | 13.48% | 6.74% | 30.34% |
| AsCOMT24 | 48.88% | 13.76% | 7.87% | 29.49% |
| AsCOMT25 | 47.15% | 13.28% | 6.23% | 33.33% |
| AsCOMT26 | 49.52% | 14.05% | 6.19% | 30.24% |
| AsCOMT27 | 46.58% | 12.88% | 5.75% | 34.79% |
| AsCOMT28 | 47.40% | 12.60% | 6.03% | 33.97% |
| AsCOMT29 | 46.99% | 13.66% | 5.46% | 33.88% |
| AsCOMT30 | 51.19% | 11.95% | 6.48% | 30.38% |
| AsCOMT31 | 45.55% | 14.02% | 6.20% | 34.23% |
| AsCOMT32 | 48.06% | 14.72% | 6.39% | 30.83% |
| AsCOMT33 | 49.45% | 12.98% | 5.80% | 31.77% |
| AsCOMT34 | 49.17% | 14.17% | 6.39% | 30.28% |
| AsCOMT35 | 49.32% | 13.35% | 7.08% | 30.25% |
| AsCOMT36 | 45.86% | 14.92% | 6.35% | 32.87% |
| AsCOMT37 | 51.53% | 9.54% | 6.11% | 32.82% |

Table S3 Primers used in this study for qPCR analysis.

| Gene Name | | ID | Primer forward sequence (5'–3') | Primer reverse sequence (5'–3') |  |
| --- | --- | --- | --- | --- | --- |
| AsCOMT24 | XM-051351214.1 | | AGGCACCAAGATCATCGCAA | ATTATCATGACCAGCGCCGT |  |
| AsCOMT32 | XM-047219123.1 | | CGGGAGTGCAGTGACATCTT | CGGTGCATTTAAGATGCGGG |  |
| AsCOMT30 | XM-047222467.1 | | TAAGGCATGTTGGGGGTGAC | AGTTCTTGAGGATCGCCGTG |  |
| AsCOMT28 | XM-051344600.1 | | CCGGATCCCTGCATCTTCAA | GCTCTCCTTGATGGCGATGT |  |
| AsCOMT29 | XM-051328539.1 | | TCTAACGCAGTGCAGGAAGG | ACCCGGCGTCCATGAATATC |  |
| AsCOMT14 | XM-047188691.1 | | GCCGGTGGGAAGGTGATAAT | TTGTACTCACTGAACCCGGC |  |
| AsCOMT23 | XM-047205743.1 | | AGTGCGTGAAGATACTGCGG | TCATCATCACGTCCCACAGC |  |
| AsCOMT8 | NM-001137149.1 | | GGGTCTTGCATGATTGGGGT | TGCTTTGAGTTTTGCGGTCC |  |
| AsCOMT1 | JQ071450.1 | | AATGCACCGTGCTAGACCTC | ACTTGAGTAGAACCGCGTCG |  |
| AsCOMT25 | OV702300.1 | | CTCCTGAGTGAACATGGCGA | CATCCCCAGCGACAAATTGC |  |
| AsCOMT22 | XM-047232741.1 | | CATTCGAAAAGGCACACGGG | ATCGTTGAATCCCGCGTAGG | |
| AsCOMT10 | XM-051353941.1 | | CAAGTGGATTTTGCACGGGT | ATCACGATCACTCTTCCGCC | |
| AsCOMT26 | XM-047231828.1 | | CCTTCCCGCACATCAAATGC | TTGACCAAGACAGCCTGAGC | |
| AsCOMT27 | JN837484.1 | | TCCTCCATGACTGGACCGAT | CTTTGTGCTTCAGGCGTAGC | |
| Actin |  | | GAGCGGGAAATTGTAAGGGAC | ATGGATGGCTGGAAGAGGAC | |

Table S4 The prediction of the interaction network of AsCOMT proteins based on the interactions of their orthologs in *Oryza sativa* L*.*

| Node1 | Node2 | Node1_string_id | Node2_string_id | Phylogenetic_  cooccurrence | Homology | Database_  annotated | Combined_  score |
| --- | --- | --- | --- | --- | --- | --- | --- |
| A0A0P0Y9M0 | Q8RV92_ORYSJ | 39947.A0A0P0Y9M0 | 39947.Q8RV92 | 0.085 | 0.91 | 0.491 | 0.514 |
| A3CH16_ORYSJ | Q8RV92_ORYSJ | 39947.A3CH16 | 39947.Q8RV92 | 0.087 | 0.906 | 0.491 | 0.515 |
| ASMT1 | Q8RV92_ORYSJ | 39947.Q6EPG8 | 39947.Q8RV92 | 0.087 | 0.907 | 0.491 | 0.515 |
| ASMT2 | Q8RV92_ORYSJ | 39947.Q8VWJ6 | 39947.Q8RV92 | 0.087 | 0.907 | 0.491 | 0.515 |
| Q2QS38_ORYSJ | Q8RV92_ORYSJ | 39947.Q2QS38 | 39947.Q8RV92 | 0.084 | 0.912 | 0.491 | 0.514 |
| Q2R333_ORYSJ | Q8RV92_ORYSJ | 39947.Q2R333 | 39947.Q8RV92 | 0.092 | 0.897 | 0.491 | 0.518 |
| Q2R6G9_ORYSJ | Q8RV92_ORYSJ | 39947.Q2R6G9 | 39947.Q8RV92 | 0.095 | 0.891 | 0.491 | 0.519 |
| Q6Z9P5_ORYSJ | Q8RV92_ORYSJ | 39947.Q6Z9P5 | 39947.Q8RV92 | 0.085 | 0.911 | 0.491 | 0.514 |
| Q75IK0_ORYSJ | Q8RV92_ORYSJ | 39947.Q75IK0 | 39947.Q8RV92 | 0.083 | 0.915 | 0.491 | 0.513 |
| Q75IK1_ORYSJ | Q8RV92_ORYSJ | 39947.Q75IK1 | 39947.Q8RV92 | 0.084 | 0.912 | 0.491 | 0.514 |
| Q8RV92_ORYSJ | A0A0P0Y9M0 | 39947.Q8RV92 | 39947.A0A0P0Y9M0 | 0.085 | 0.91 | 0.491 | 0.514 |
| Q8RV92_ORYSJ | A3CH16_ORYSJ | 39947.Q8RV92 | 39947.A3CH16 | 0.087 | 0.906 | 0.491 | 0.515 |
| Q8RV92_ORYSJ | Q2QS38_ORYSJ | 39947.Q8RV92 | 39947.Q2QS38 | 0.084 | 0.912 | 0.491 | 0.514 |
| Q8RV92_ORYSJ | Q2R333_ORYSJ | 39947.Q8RV92 | 39947.Q2R333 | 0.092 | 0.897 | 0.491 | 0.518 |
| Q8RV92_ORYSJ | Q2R6G9_ORYSJ | 39947.Q8RV92 | 39947.Q2R6G9 | 0.095 | 0.891 | 0.491 | 0.519 |
| Q8RV92_ORYSJ | Y1103_ORYSJ | 39947.Q8RV92 | 39947.Q53QK0 | 0.091 | 0.898 | 0.491 | 0.517 |
| Q8RV92_ORYSJ | ASMT1 | 39947.Q8RV92 | 39947.Q6EPG8 | 0.087 | 0.907 | 0.491 | 0.515 |
| Q8RV92_ORYSJ | Q6Z9P5_ORYSJ | 39947.Q8RV92 | 39947.Q6Z9P5 | 0.085 | 0.911 | 0.491 | 0.514 |
| Q8RV92_ORYSJ | Q75IK0_ORYSJ | 39947.Q8RV92 | 39947.Q75IK0 | 0.083 | 0.915 | 0.491 | 0.513 |
| Q8RV92_ORYSJ | Q75IK1_ORYSJ | 39947.Q8RV92 | 39947.Q75IK1 | 0.084 | 0.912 | 0.491 | 0.514 |
| Q8RV92_ORYSJ | ASMT2 | 39947.Q8RV92 | 39947.Q8VWJ6 | 0.087 | 0.907 | 0.491 | 0.515 |
| Y1103_ORYSJ | Q8RV92_ORYSJ | 39947.Q53QK0 | 39947.Q8RV92 | 0.091 | 0.898 | 0.491 | 0.517 |


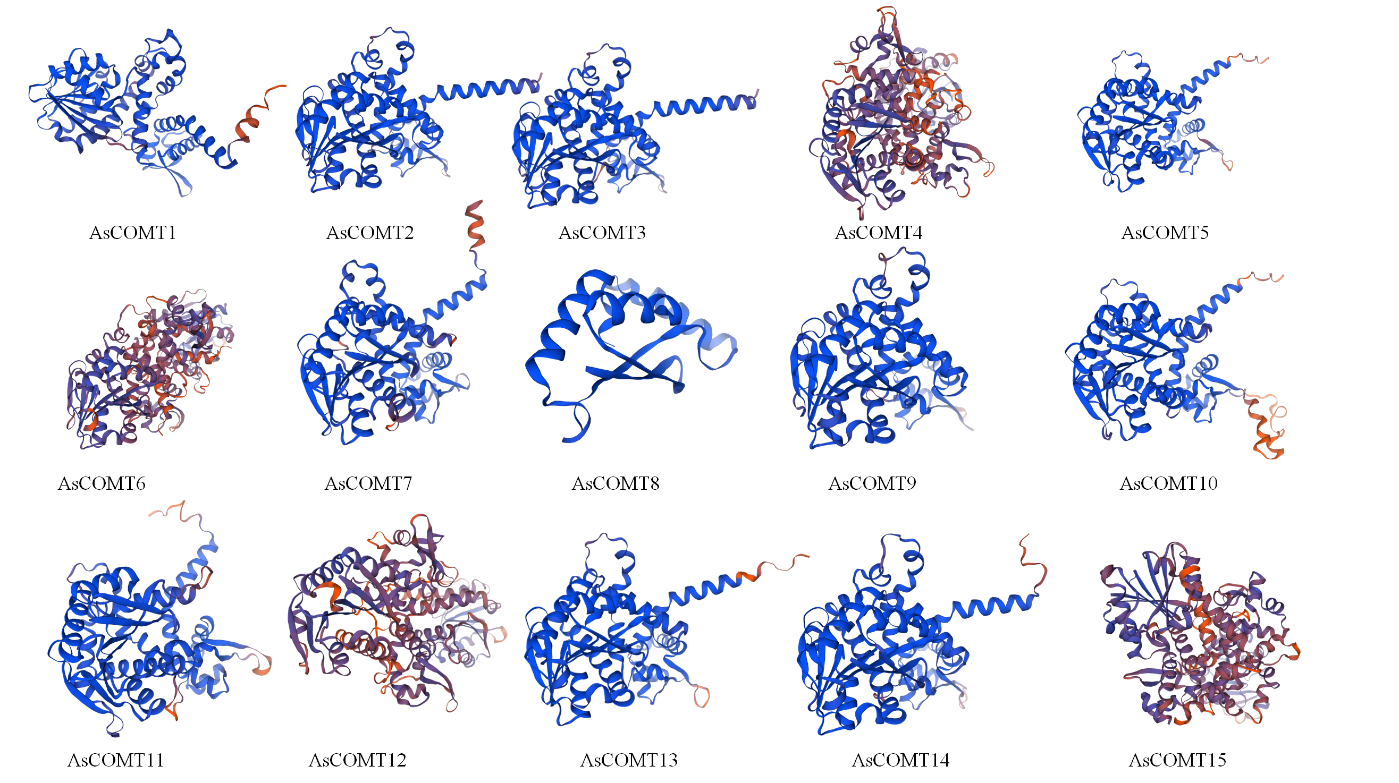


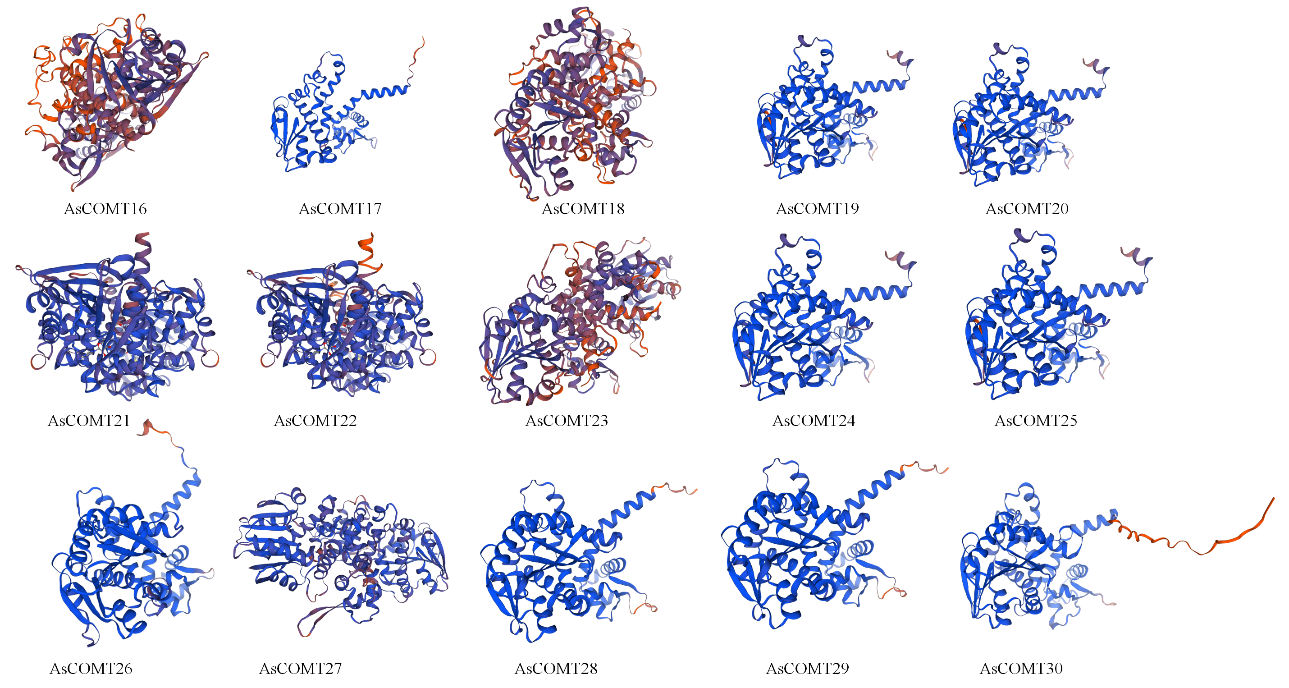


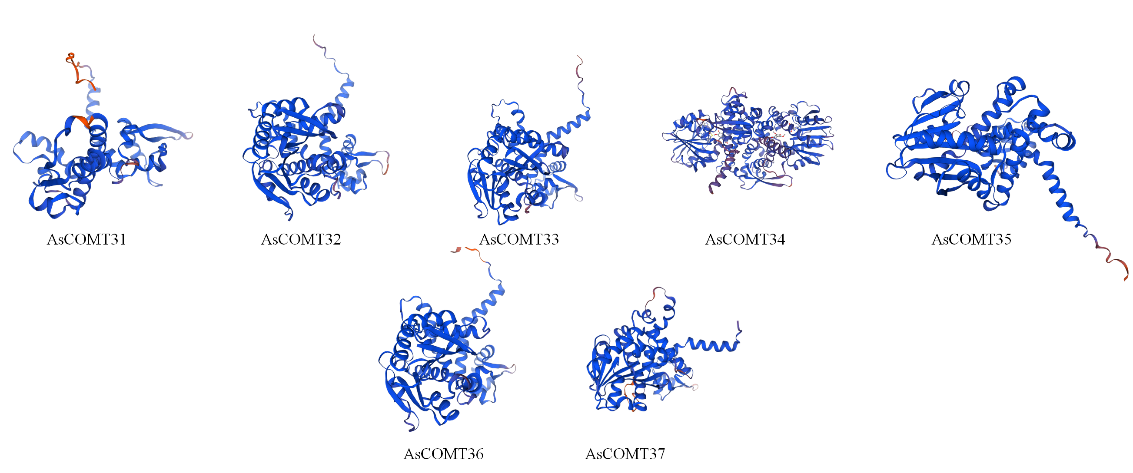


Fig. S1 Tertiary structure predictions of AsCOMT proteins. Blue indicates α-helices, green indicates β-folds, yellow indicates irregular curls, and red indicates extension chains.


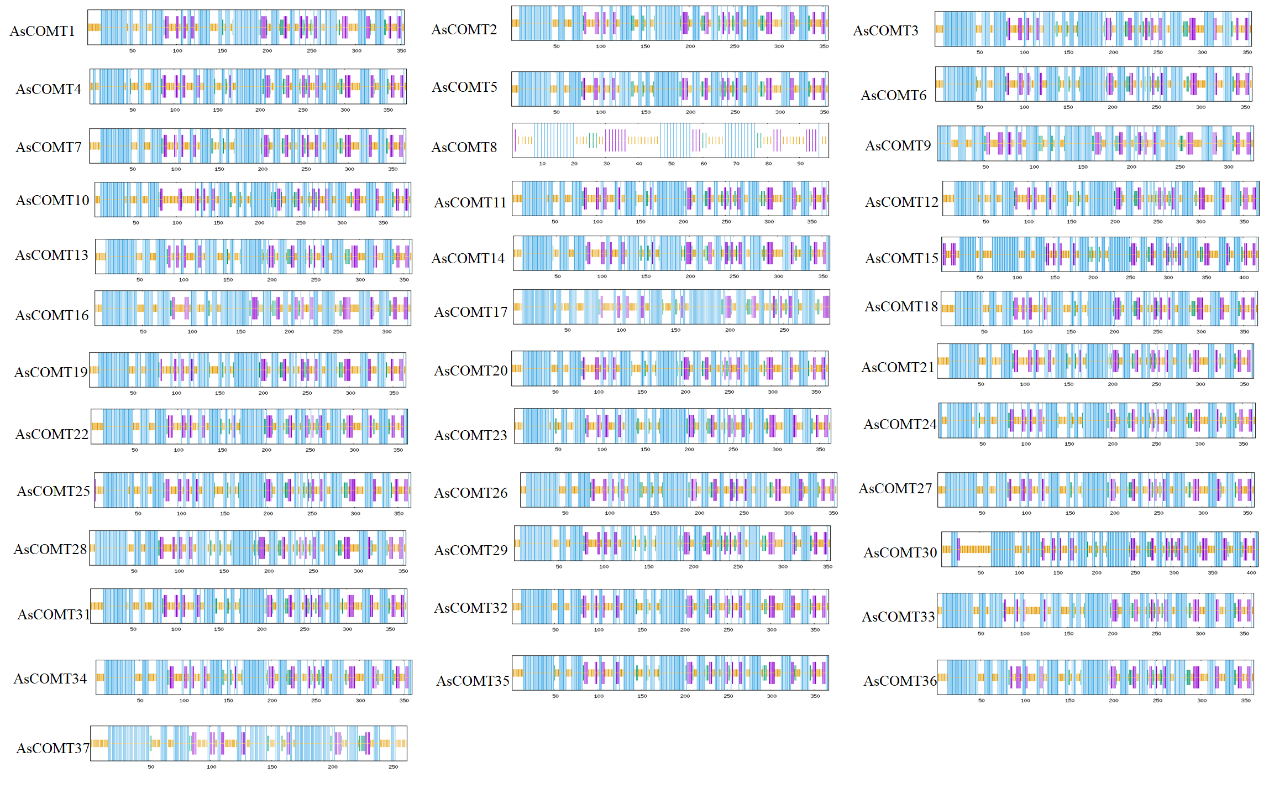


Fig. S2 Protein secondary structure encoded by AsCOMTs in *Avena sativa*.

Note: Blue represents α- Spiral. Red indicates irregular curls.


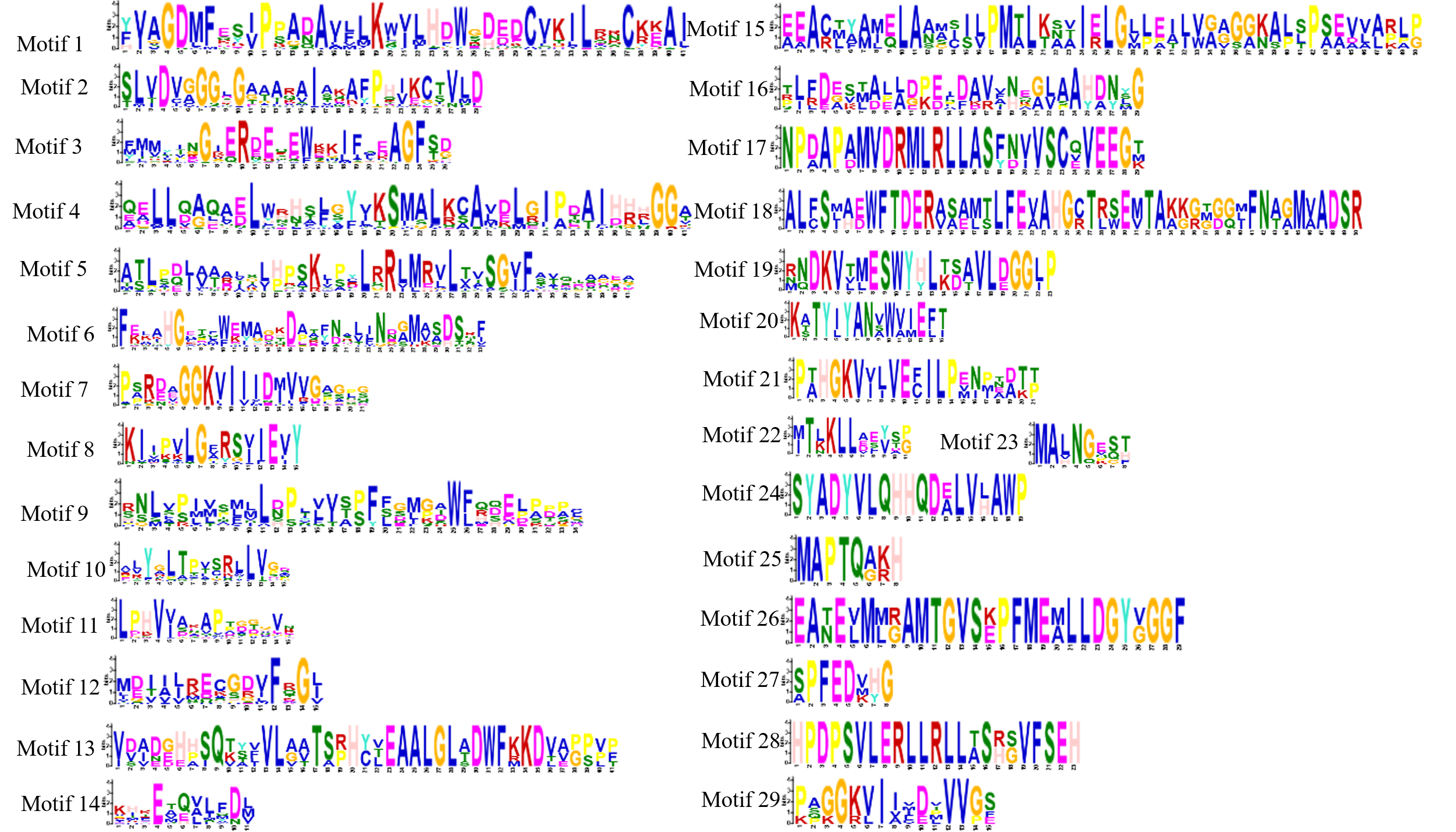


Fig. S3 Sequence marker soften motifs of AsCOMTs protein identified by MEME program.

Note: Each site is displayed in alphabetical (amino acid residue) order. The total height of the amino acid stacking represents the information content of the site in the motif. The height of each letter in the stack represents the degree of conservation of the amino acid in that position. The X axis is the length of the motif, and the Y axis is the site of the letter.


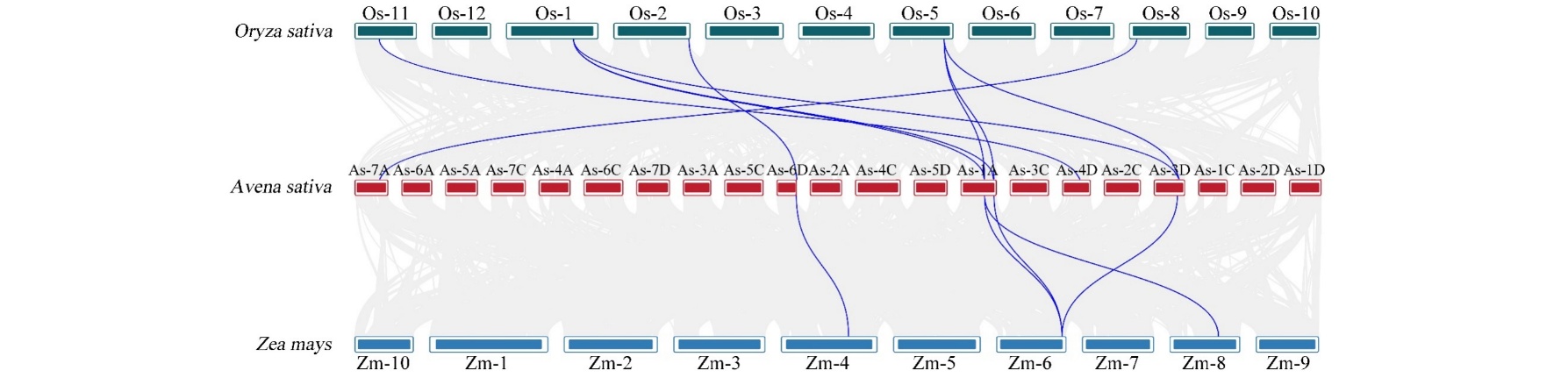


Fig. S4 Collinearity analysis of the *COMT* genes among oats, *Z. mays*, and *O. sativa*. Gray lines in the background indicate syntenic genomic blocks between oats and other species, while blue lines highlight the syntenic *COMT* gene pairs.


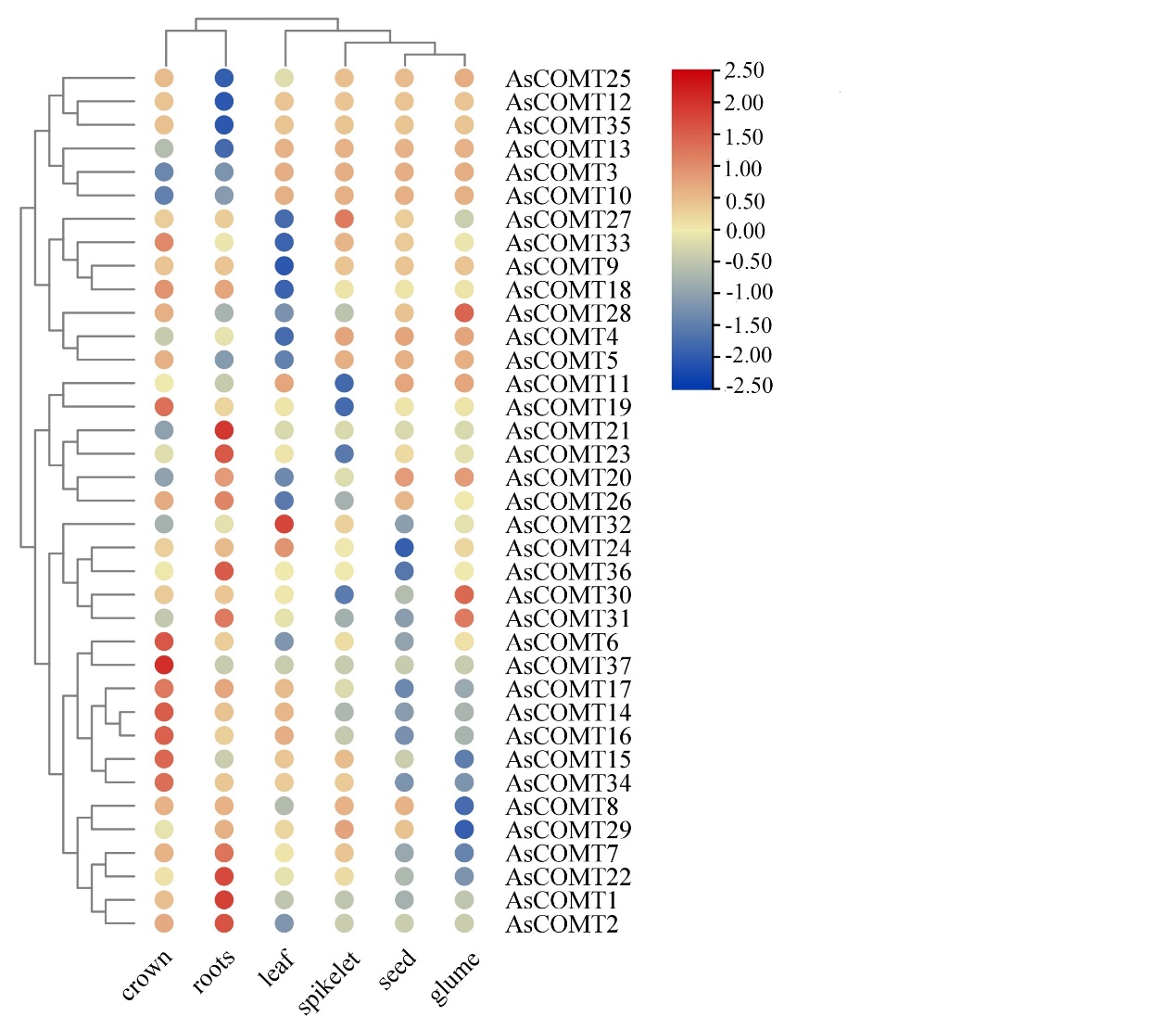


Fig.S5 A heat map of oat tissue-specific gene expression of AsCOMT genes.


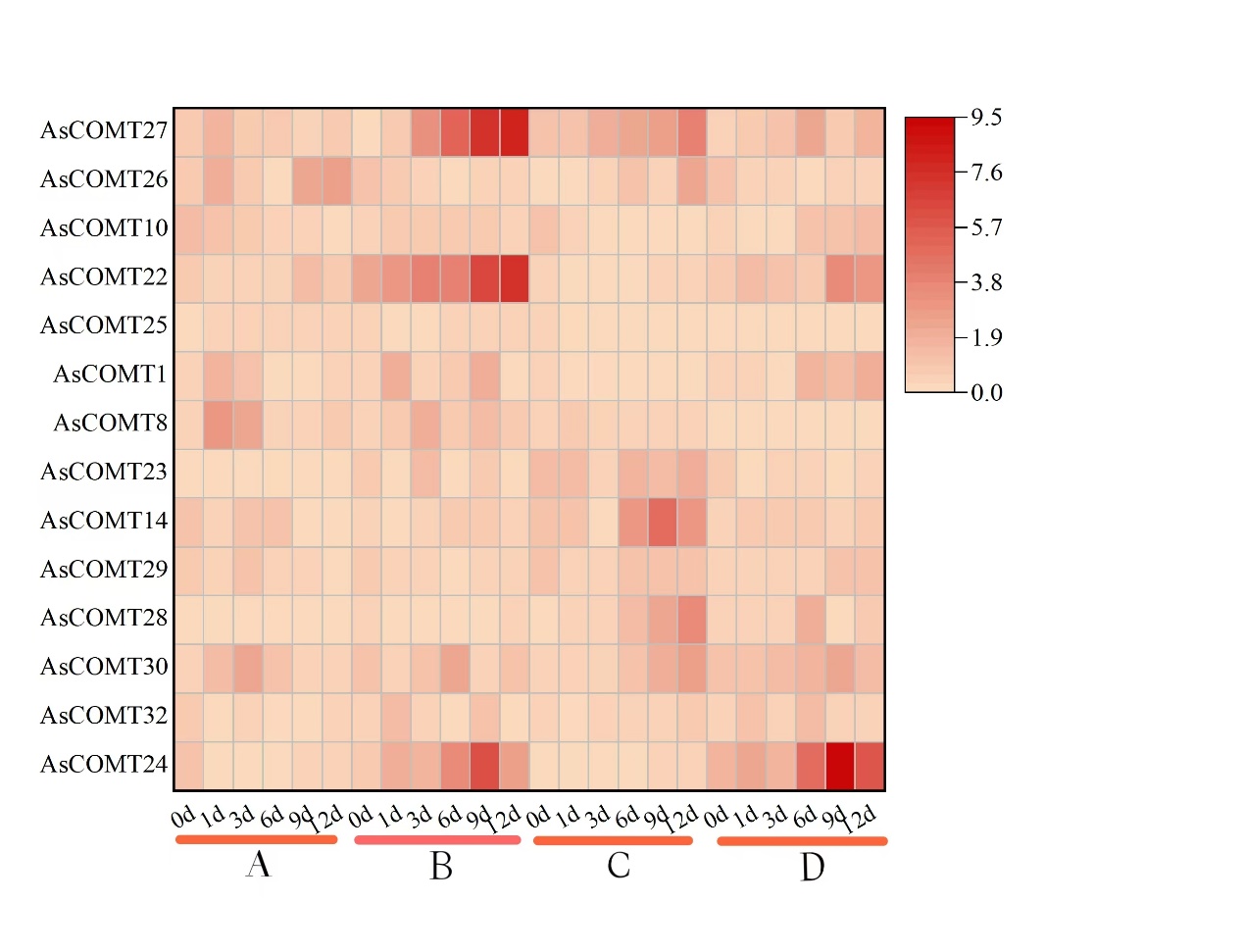


Fig. S6 The AsCOMT gene expression heatmap after infection with powdery mildew and leaf spot in two oat varieties.

A: The oat variety 'ForagePlus' is infected with powdery mildew. B: The oat variety 'Molasses' is infected with powdery mildew. C: The oat variety 'ForagePlus' is infected with leaf spot. D: The oat variety 'Molasses' is infected with leaf spot.
