# Supplementary material for: Genome-wide analysis of the COMT gene family in Avena sativa: insights into lignin biosynthesis and disease defense mechanisms
Source: Front Plant Sci. 2025 Jun 19;16:1609698. doi: 10.3389/fpls.2025.1609698 (PMC12222199; doi:10.3389/fpls.2025.1609698)
Supplement: Supplementary file 2 [file Table1.docx]

The accession ID and protein sequence of the COMT gene of the species used obtained from NCBI

*Avena sativa L.*

>AsCOMT24 Accession ID: KAK1678936.1

MTLKLLVEVSPQELLVSLAELQNHILGYVKSMSLKCAVDLDIPGAIHRRGGTATLADIVIDTKVHPAKVADLQRVMELLSASGIFSTTAAGEDGGAVLYGLTTTCRFLVGWRNLSPMVPFLANPLVVSSFSSMPDWLRSEPAPAGAGSLFELAHGCSQWEMGSKDAEFNSVMNSSRAADNQLFLEVIIFDKGRIFRGLSSIVDVGAAKGEGTKIIASAFPRIKCTVMDLPHVVGQDADGAGHDNLQFVAGDMFQSIPSADAVVLKNILHDYGHDDCVKILQRCKEAIPAKNVGGKVIIIDMVRGSAHGDMKISEMEALQNMYMMYTNGMERDESEWKRIFSDAGFSDNYKIMPVLGPYSVIEIYP

>AsCOMT32 Accession ID: XP_047075078.1

MSTRAIEVPTDAELLQAQADLWRHSLYYLASMGLRCAVELEIPTTIHHLGGVTSLPDLMTALSLPTVKMPFLGRLMRVLVNSGVFASDNNSKSGEELFRLTPLSRVLVHGVVANEHHSQKYFVLGVTSPHYTEAALGLADWFKKDTVPPVTSPFEDKYGVPLFDEKTALLDKELDDVVNKGLAAHDNLGIGTILRECSDIFKGVESLTDCCGGDGTTARALVKAYPHLKCTVLDLPKVIEKAPPHDVINYVAGDLFHTVPPSQAVMLKLVLHFWSDEDCVKILAQCRKAIPLREEGGKVIIIEIVVEPSLGPIMFEAQLLFDMLMMVNTRGGQRDEKHWRELFMQAGFTDYKIVKKLGARSVIEVYP

>AsCOMT30 Accession ID: XP_048537012.1

MSFPHAHQTYSTYKADRLLDNSRTTRGTRRLTSLARSVHQSTSLKMSSPATDPTSAAADDLSPAEARLAMMELANMISVPMALTAVIRLGVPAAIWAGGANAPLPAADLLPPGHPDPSVLERLLRLLASRGVFSEHTAAPGAARRYALTAVGRTLVPTGPSGASYADYVLQHHQDALVLAWPRLRDAVLDPAGPEPFARAHGGVPAYAYYGQDREANEVMLRAMTGVSEPFMEALLDGYGGGFDGVATLVDVGGSSGACLEMIMRRVGTIREGVNFDLPDVVAAAPPIAGVRHVGGDMFKSIPTGDAIFMKWVLTTWTNDECTAILKNCHEALPQGGKLIACEPVVPETTDTSTRTRALLENDIFVMTTYRTQGRERSEEEFRQLGLTAGFTGFRALYLDPFYAVLEYVK

>AsCOMT28 Accession ID: XP_051200558.1

MALIHSTNQALLDGQLNLLDNTFGYIKSMALKAALDLRIADAFDHHGGAATLTQIADRVTLHRSKISSLRRLMRVLIVSGVFGVQSAEGSSELLYVLTPTSSLLVGPRNLVSITTMSLSPYFVASYLELGTWFHQELPDPCIFKLAHGEPLWKLAEHDASFDALINDGMVSDTSFIMDIAIKESGGVFQGITSLIDVAGGLGAATQAISKEFPHVEYTVLDLDHVIAKAPTGTNVKYIAGDMFESIPRANAVFLKWILHDWGHDDCVKILRNCKKAIPPRDAGGKVIILDIVFGAGQSNVKHTEVQASFDVYMMIINGIERDELEWKMMFSEAGFTDYKIIPVLGFRSIIEVYP

>AsCOMT29 Accession ID: XP_051200558.1

MALIHSTNQALLDGQLNLLDNTFGYIKSMALKAALDLRIADAFDHHGGAATLTQIADRVTLHRSKISSLRRLMRVLIVSGVFGVQSAEGSSELLYVLTPTSSLLVGPRNLVSITTMSLSPYFVASYLELGTWFHQELPDPCIFKLAHGEPLWKLAEHDASFDALINDGMVSDTSFIMDIAIKESGGVFQGITSLIDVAGGLGAATQAISKEFPHVEYTVLDLDHVIAKAPTGTNVKYIAGDMFESIPRANAVFLKWILHDWGHDDCVKILRNCKKAIPPRDAGGKVIILDIVFGAGQSNVKHTEVQASFDVYMMIINGIERDELEWKMMFSEAGFTDYKIIPVLGFRSIIEVYP

>AsCOMT14 Accession ID: XP_047044647.1

MAPTQGKHSSQDLLQAQVDLWHHALGFVKSMALKCAMEMQIPNTIQHHGGAMTPSELATAIGLHPSKLPRLRRLMRVLTVSGIFVVHESASADKEAVYGLTPTTCLLVSDEVKSNLFPILSLMLDSTVITPFFGMKSWFQDEDSTSLFKKIHGLTFWEMADQDETYNQLINNAMVSDSNFLMDIILRECGDVFTGISSLIDVAGGLGGAARAIAKAFPQIKCTVLDLPHVVADAPTDDHVPFIAGDMFEYIPPANALFLKWIFHDWGDEDCVKILKKCKEAIPSRDAGGKVIIVDMVVGSGPNDIVTRETQVFFDLFIMYLEGIEREEFEWKKIFMEAGFSEYKIISVLGVRSVIELYP

>AsCOMT23 Accession ID: XP_047061699.1

MDADRDPSLATDDELLRAQAELWSHVFAYTKSMALRCAVELGIPDAVHRRGGSATVPDLVAELSLPPSRAPYLRRLLRLLAHAGFFDAAGSEEPEDTYGLTLLSRLLVSAPGAGQGLSPFALAMLHPVIVSPSMSLASWFRAADGSARVPFAAAHGGRELWAVAKDDPGFGAAFNDAMACDGRFVMDLIVRGNGRDLFRELASLVDVGGGSGGAAKAIAAAFPHVRCSVLELPHVVASVPPGDGGVEFVAGDMFEHVPKADAVLLKWILHGWGDEECVKILRRCKEAVPAGGRVIVMDLVVGSSPGDARATETQLLWDVMMMGVVGSPERDEREWSKIFHDAGFSGYKILSILGIRSVIEVYP

>AsCOMT8 Accession ID: XP_066367447.1

WVLHDWGDAECVKILKNCKRAIPSRDDGGKVIVIDMVVGAGPQNSKHKDTEVMFDLFIKLFNGIERDEQEWKKITFEAGFSDYKVIPALGVRSIIEVYP

>AsCOMT1 Accession ID: AFU52936.1

MGHVHTTSENKAMMSTEEFLQAQTELYNLSLAYVKSVALRASIDLQIPEAIHRRGGSATLSDIAAETGTHGTKVSYLGRLMRVLAISGVFSMHSDDGAVYYKLTHVSRLLVPALSPMVPVLVDPLAATALFSLADWFTDERVSELTLFEAAHGCTRSEMTAKKGMGGMFNAGMVADSRVLTDILLSEHGDMFEGVESLVDIGGGHGGVAEAIAKALPNMKCTVLDLPHVVKEAATGNVQFVAGDAFQYIPPADAVLLKWFLQLFNDEDAIKVLRRCREAIPAKGKVIIFDVVVGSNCKDGPTRETQLLFDIFMMRVGGREREEQQWRKIIFEAGFTDYKISVVLGFRSIIEVYP

>AsCOMT25 Accession ID: XP_047083194.1

MTLKLLAEVSPQELLVGLAELHNHFLGYVKSLSLKCAVDLGIPEAIHRRGGTATLADIATDTKVHPAKVADLRRVLELLTASGMFTTSTAGEDGGGAVLYGLTTTCRFLVGWHNLSPMVPFLLNPLVVSSFFSMPDWLRSEPAPAAAGSLFELAHGCSQWEMATKDAEFNDVLNGSMAADSQVFLEVIIMDKGRIFRGLGSLVDVGGGKGAGTKVIASAFPRIKCTVMDLPHVIAGQVDAGHENLNFVAGDMFQSIPSADAVVLKNILHDWGHDDCVKILQRCKEAIPARNAGGKVIIIDMVRGLAHDEKKLTEMEAIQNLFMMYINGVERNESEWKGIFSDAGFSDDYKIMPVLGPYSVIEIYP

>AsCOMT22 Accession ID: XP_051178062.1

MGSTAEMFVVPAVTNDEEACTYAIELAAACILPMTLKSTIELGLLEILVGAGGKALSPSEVVARLPSGTSNPDAPAMVDRMLRLLASFNVVSCQVEEGTDGLLARRYSPAPVCKYLTPNEDGVSMAPLVLARNDKVTMESWYHLTSAVLDGGLPFEKAHGMPAFKYYGTDARFNRVFNEAMKSHSTIITKKLLDSYAGFNDIGTLVDVGGGTGATICAITSKYPHIKGINLDLPHVISEAPPSPGVRHVCGDMFKEVPSGDAILMKWILHDWTDEKCLTLLRKCYDALPTHGKVILVEFILPENITDTTKGVFMADMAMLTNTPGGKERYRREFEALANCTGFGAFKATYIYANVWVIELTK

>AsCOMT10 Accession ID: XP_051209901.1

MAVNGVSTDQALLDAEHELWRTSFSYIKSMAVKSALDLRLADAIDHHGGAATLPQIVARVAVHPSKVPCLRRLMRVLAVSGVFSVQQHQTVLPAAIANDNGNGAVITNGVGAATANGNGTAAEPLYALTPVSRLLVGSRSLASIMSMILDPAFITPFLGIGAWFEHPLPDPCIFRQQHGVALWKMADKDPAFDALVNDGMVSDSSFIMEIAIRECGEVFQGITSLIDVAGGLGAASQVISKAFPGLECTVMDLGHVIAKAPSSTAVKYVVGDMFESIPPADAVFIKWVLHDWGHKDCVKILKNCKKSIASGEKGGKIIIMDIVVGARPSGEKHKELQVLFDMYMTIVDGIERDEHEWEKIFVEAGFSGYKIIPVLGFRSIIEVYP

>AsCOMT26 Accession ID: XP_051178062.1

MAAQAQLTVPTDAELLQAQADLWRHSLYYMTSMAFQCAVKLGIPTTIHRLGGTASLPDLVTALSLPPAKLPYLRRIMRLLATSGVFATTDVDVYRLTPISFLLLDGVDVDGHPSQTTVVLAATARHCVEAALGLTDWFRKDVAGSPFEDLHGVTLFDGSMAEAEPEIDAVFNEALAAHDNSGFVAVLRECGGTLFQGLDSLTDCCGGDGTSARSIVEAFPHIKCTVLDLPRVIDAIPADGVVNYVAGDMFNLVPPAQAVLVKLVLHHWSDEDCVKILAQCKKAIPSREEGGKVIVIDILVDPSSGPTHEAELLVDVAMMVLTNGRQRDESDWAEIFTKAGFTGYNIVKKLGARGVFEVYP

>AsCOMT27 Accession ID: KAK1607286.1

MGSITTATIADDEAACMYAMQLAAGSILPMTLKNAIELGMLEILVGAGGKALSPSEVVARLPFAATNPDAPAMVDRMLRLLASFDVVSCEVEEGMGGVLARRYGPAPVCKWLTPNKDGVSIAPLVLMNDKVVLESWYHLKDTVLEGGLPFERAHGVTAFEYHGTDPRFNHVFNEAMKNHSTIITNKLLEFYTGFDDIGTLVDVAGGVGATIGAITSKYPHIMGVNFDLPHVLSEAMPFPRVQHVGGDMFKKVPSGDAILMKWILHDWTDEQCVTLLRNCFDALPAHGKVVIVECILPMIPAATPEAQRSFEFDMIMLTHTPGGKERDQREFEELSKSAGFTSVKTTYIYANSWVMEFIK

>AsCOMT13 Accession ID: XP_047045402.1

MALNGQSTDQALLDAEQELWTTSFSYIKSMALKSALDLRLADAIDHYGGAATLSQIVARVTLHPSKIPCLRRLMRVLTLSSVFTVQQAAGAGDPLYALTPVSRLLVGSRSSASLMACVLNPVLVTPFLQIGAWLQHALPSPCIFEHTHGEGIWKMAGKDAAVDSLINNALASDSHFIVDIAVKEAGDVFRGLSSLVDIGGGLGEAAQVISEAFPHVECSVLDLEHVVSNAPAGTNVKYVAGNMFESVPPANAAFLKSVLHDWDDEKCVKILESCRKAIPPREVGGKVIIIDIVVGAGLGDNKHKEVHALFDMYIMLINGIERDEQEWSKIFLEAGFSDYKITPVLGFRSIIEVYP

>AsCOMT12 Accession ID: XP_047093062.1

MALNGPSTDQALLDAEHELWTTSFSYIKSMALKSALDLRLADAIDHHGGATTLPQIVARVMLHPSKIHCLRRLMRVLTLSSVFTTQQADGTGEPLYTLTPIGAWFQHALPDPCIFEHMHGEALWEMAGKDATLDALINKAMVSDSGFIMDVAVREAGDVFRGLSSLVDVGGGLGAAAQVISKAFPHVGCSVLDLEHVVSNAPAGTNVKYVAGDMFESVPPANAAFLKSVLHDWDDEKCVKILESCRKAIPPREVGGKVIIIDIVVGAGLGDEKHKEVHALLDMYIMFINGIERDEQEWSKIFLEAGFSDYKIIPVLGFRSIIEVYP

>AsCOMT11 Accession ID: XP_047045402.1

MALSSDIYKLMSTEDMLQGHAELCIHAYGFVKSMALKCAMELGIPGAIHGNGGCATLGELATRIALPPSRLPRLRRLMRVLAVSGVFSVQHQPDSAGDALYGLTVASRLLVVDNEETSSSGLTRLVSLILDPNLTAPFSGMSAWFMDDERQRSFFEMHHGEDMWDMASRDSALTRTIGDGMAADSRFVVEVLLRESRARDVFRGVRSMVDVGGGSGAIARAIAAAFPHVECSVLDLPHVVAEAPEDGDVRFVSGDMFEYIPPADAVLLKSVMHDWRDDECVKILRRCKEAIPSRDAGGKVIIINMVVGSESSNQGNDRKEEAQVLYDLFLMVVEGSEREEHEWEKIFLEAGFSGYKIIPVLGIRSIIEVYP

>AsCOMT9 Accession ID: XP_047045430.1

MALKSALDLGIADAIHHHGGAATLPQIVAKAALHPSKLSCLSRLMRVLTVSGIFNIQDAQNGDEAVYTLTSASRLLVGTPSLVPALHMMLHPIQVSSFFDFGKWFQQELPEGMDLFTLKHGKTMWELADQDPAYNALLNNGMVSDSRFLMDIVLKECSDVFRGISSLVDVAGGLGGAAQTISKAFPHVKCSVMDLAHVVAKAPTGTDVEYIVGDMFDSVPPANVVFLKWVFHDWGDAECVKILKNCKRAIPSRDGGGKVIVIDMVVGDGPQNSKHKESQVIYDLFIKLINGIERNEQEWRKIIFEAGFSDYKIIPALGVRSIIEVYP

>AsCOMT7 Accession ID: XP_047045335.1

MAALIQPDELAMSADELLQAQVELYHHCFAFVKSLALKAATDLGIPDAIHRRGGAATLSDDLAAETGIHPTKLSSLRRLMRVLTTSSIFSIVDDAGGAVYKLTRVSRLLVGAGGRAHLSPAVGAFVSPFPVAALFSMHEWFTDERAAAMSLFEVAHGRTLWEVTAAGRGDQIFNTAMAADSRFTMESLLREECGGAVFGAVRGSLVDVGGGHGATASAIARAFPHVKCTVLDLPHVVAEAPVDDTLTFVAGDMFDHIPPADAVLLKWILHEWNHEDCVKILRRCKEAIPTQDEGGKVIIIETIIGSPGSQGIVSTENEVLLDGFMMCMDGIEREDQEWSKIFFEAGFTDYKITTTTGFRSIIELYP

>AsCOMT6 Accession ID: XP_047044647.1

MAPTQAKHSSQDLLQAQVDLWHHALGFVKSMALKCAMEMQIPNTIQHHGGAMTPSELATAIGLHQSKLPRLRRLMRVLTVSGIFVVHESASADKEAVYGLTPTTCLLVSDEVKSNLFPILSLMLDSTVITPFFGMKSWFHDEDSTSLFKKIHGLTFWEMADQDETYNQLINNAMVSDSNFLMDIILRECSDVFTGISSLVDVAGGLGGAARAIANAFPQIKCTVLDLPHVVADAPTDDHVPFIAGDMFEYIPPANALFLKWIFHDWGDEDCVKILKKCKEAIPSRDAGGKVIIVDMVVGSGPNDIVTRETQVFFDLFIMYLEGIEREEFEWKKIFMEAGFSEYKIISVLGVRSVIELYP

>AsCOMT5 Accession ID: XP_051209879.1

MALNGKSTDQALLDAEQELWTTSFSYIKSMALKSALDLGLADAIDHYGGAATLSQIVARVTLHPSKIPCLRRLMRVLTLSGVFTIQQADGAGDPLYALTPVSRLLVGSRSSASLMACVLSPVLVTPFLQIGAWFQHALPNPCIFEHTHGEAIWQMAGKDGTVDSLINNALASDSQFIVDIAVKEAGDVFRGLSSLVDIGGGLGEAAQVISEAFPHVECSVLDLEHVVSNAPAGTKVKYVAGDMFESVPPANAALLKSVLHDWDDEKCVKILESCRKAIPPREVGGKVIIIDIVVEAGLQDKKHKEVHVLFDMYMMLINGIERDEQEWSKIFVEAGFSGYKITPVLGFRSIIEVYP

>AsCOMT4 Accession ID: XP_047093062.1

MALTGDIYKLMSTEDMLQGHAELCIHAYGFVKSMALKCAMELGIPAAIHGNGRGGGATLGELANRIALPPSRLPRLRRLMRVLTVSGVFSLQHQPEPAGDAVYGLTVASRLLVGDNDETSSSGLTRLVSLMLDPNLTAPFSGMSAWFMDDERPRSFFEMHHGEDMWDMAAREAALSSTIGDGMTDDSRFVVEVLLRESRACDVFRGLWSMVDVGGGSGAIARAIAAAFPHVECSVLDLPHVVAEAPEDGDVRFVSGDMFEYIPPADAVLLKSVMHDWRDDECVKILQKCKEAIPSRDEGGKVIIINMVVGSGTSNQGKDLKEEAQVLYDLFLMVFEGGEREEHEWEKIFLEAGFSGYKIIPVLGIRSIIEVYP

>AsCOMT3 Accession ID: XP_047069291.1

MALNQEQHILDQGLLDGQLVLWHNTFSYIKSMALKSALDLGIADAIHHQGGKASLPQIVAKATLQPSKISCLRRLMRVLTVSGVFATEHSADGEAVYALTPASRLLVGSVNMVPIMNMLLHPILVSPFSDLGTWLQQELPDPELFKLKHGKTFFELADHDPSYNKLLNDGMASDSRFLMDIAIRECPGVFQGIGSLVDVAGGHGGAAQAIAKAFTDVKCSVLDLDHVVAKAPTGTAVEYIAGDMFESVPPADAVFLKWIMHDWRDADCIKILKNCKKAITAKDIGGKVIIIDMVVGAGPQDLKHKETQVMFDLFIMFINGIERDEQEWKKIIFEAGFSDYKITPVLGVRSIIEVYP

>AsCOMT2 Accession ID: XP_047045403.1

MALNGEQHILDQGLLDGQLELWHNTFSYIKSMALKSALDLGIADAIHHQGGKATLPQIVAKTALHPSKISCLRRLMRVLTVSGVFATEHSADGEAVYALTPASRLLVGSANMVPIMNMLLHPILISPFSDLGTWFQQELPDPDLFKLKHGKTFWEMADHDPSYNTLVNDGMASDSRFLMDIAIRECAGVFQGIGSLVDVAGGHGGAAQAISKAFPDVKCSVLDLDHVVAKAPTGTAVEYIAGDMFESVPPADAVFLKWVMHDWSDVDCIKILKNCKKAIAAKDVGGKLIIIDMVVGAGPQDPKHKETQVMFDLFIMFVNGIERDEQEWKKIIFEAGFSDYKITPVLGVRSVIEVYP

>AsCOMT16 Accession ID: XP_037489468.1

MDSHGKPIPATSAEEELVQAHAELMRHSFGYLKSMALSSAVKLGIPDAIHHHGGAASLPELLAILPLPQSKRPYLSRLMKMLVVAGIFGDAPVGEGCEVRYCLTPVSRLLVGDAAVNGGACRSPVVLVATSAAHLTASARLHEWLLQEEVESAFAMAHNGEGFFGVAGGEGVCSALNSEAKASESRREAETVVRACGRVFEGITSLVDVGGGNGTAARAIARAFPHVKCTVLELPHVVDAVSALPSEEEGAVEFVAGDMMQSIPPADAILFKYVLHNWSDEDCLGILTRCREAIPSGGKVIIIDTVVGFGSPSHEILESQLLMDMCMMVLLDGKERDEQCWGKIFMEAGFSHYKIKHLQALGSAIEVYV

>AsCOMT15 Accession ID: XP_047069235.1

MELAWSFTASSPPSLVCTTFARTAPCSGQLGCQRRMPGMWRSRVVRSHRQGGMVVLHAKTENDDMMSTEGLLEAQLELYHHSMAYVKSAALRAAADLRIPDAIHRHGGVATLSDVATETGIHPTKYSHLRRLMHALTIFGIFSVEGDARYKLTRVSRLLVEGGEGSCTQTTQSPSVRVLVDPLSLTALCSIAEWFTDERASAMTLFEVAHGCTRSEMTAKKGTGGLFNAGMVADSRLVMETVLQQHSSVFQGVSSLVDAGGSHGAAAAAITKFLPHIKCTVLDLPHVVAGAPTAVGGNVQFVAGDVFEYIPPADAVLLKWVMCLWQDEDAIKVLRRCKEAIPARDDGGKVIIIDAVVNTCWGSQNVLLRETQALFDVQMMRVDGCEREEHQWRNIFVEAGFRDYKITPMLGFRSIIEVYP

>AsCOMT20 Accession ID: XP_047083194.1

MTLKLLAEVSPQELLVGLAELHNNFLGYVKSLSLKCAVDLGIPEAIHRRGGTATLADIATDAKVHPAKVADLQRVMELLTASGIFTTSTAGEDGGGAVLYGLTTTCRFLVGWHNLSPMVPFLLNPLVVSSFFSMPDWLRSEPAPAGAGSLFEVAHGCSQWEMGRKDAEFNDILNGSMAADSQVFLEVIIMDKGRIFRGLGSLVDVGGGKGAGTKVIASAFPRIKCTVMDLPHVITGHVDAGHENLNFVAGDMFQSIPSADAVVLKNILHDWGHDDCVKILQRCKEAIPARNAGGKVIIIDMVRGLAHEEKKIDEMEAIQNLFMMYINGVERNECEWKGIFSDAGFSDDYKVMPVLGPYSVIEIYP

>AsCOMT19 Accession ID: XP_047083194.1

MTLKLLAEVSPQELLVGLAELHNNFLGYVKSLSLKCAVDLGIPEAIHRRGGTATLADIATDAKVHPAKVADLQRVLELLTASGIFSTSTAGEDGGGAVLYGLTTTCRFLVGWHNLSPMVPFLLNPLVVSSFFSMPDWLRSEPAPAGAGSLFEVAHGCSQWEMGKKDAEFNDTLNGSMAADSQVFLEVIIMDKGRIFRGLSSLVDVGGGKGAGTKVIASAFPQIKCTVMDLPHVIAGQVDAGHENLNFVAGDMFQSIPSADAVVLKNILHDWGHDDCVKILQRCKEAIPARNAGGKVIIIDMVRGLAHVEKKITEMEAIQNLFMMYINGVERNESEWKGIFSDAGFSDDYKIMPVLGPYSVIEIYP

>AsCOMT18 Accession ID: XP_047087786.1

MAAHHAPTMAVPTDAQLIQAQADLWRHSLCYFTPMALRCAVQLGIPTAIHRLGGTASLSDLVAALSLPPSKTPYLSRIMRLLVTSGVMASHEEGVYSLLPLSYLLVDGISIDGEASQVAIVLAATSRHYLEAALGLSDWFKKDVAPPLPAPFEDVHGATILEESMALLDPESDKVIHEAVAAHDNMGIGTILRECHDLFKGLESLTDCCGGDGTTARAIVQAFPHIKCHVLDLPKVIERAPRGDGSVNYVAGDLFHSIPAAQAVMLKLVLHFWSDEDCINILSHCKKAIPSREAGGKLIIIDIVVDPSSSGQILETQLLMDVAMLVCTRGRQRDENDWRSIFTKAGFSDYKIVKKLGARGVIEVYP

>AsCOMT17 Accession ID: XP_047044647.1

MAPTQARHSSQDLLQAQVDLWHHALGFVKSMALKCAMEMQIPNTIQHHGGAMTPSELATAIGLHPSKLPRLRRLMHVLTVSGIFVVHESASADKEAVYGLTPTTCLLVSDEVKSNLFPILSLMLDSTVITPFFGMKSWFHDEDSTSLFKKIHGLTFWEMADQDETYNQLINNAMVSDSNFLMDIILRECSDVFTGISSLVDVAGGLGGAARAIANAFPQIKCTVLDLPHVVADAPTDDHVPFIAGDMFEYIPPANALFLKWIFHDWGDEDCQDTEKMQGSYPFQRCRWEGDNC

>AsCOMT33 Accession ID: XP_044443717.1

MAAQAQAQSIEVPTDAELLQAQADLWRHSLYYLSSMGLRCAVQLEIPTAIHRLGGVTSLPDLMSALSLPSCKLPFLHRLMHVLVTSGVFAADTDSESGVELYRLTPLSRILVEGVDAEEHHSQKYFVLGVTSPHCTEAALGLADWFKKDLEPPVPSPFEDMHGVPIFDERTALLDKDFDAVTNQGLAAHDNLGIATILRECGDLFMGLESLTDCCGGDGTTARAIVKAYPHIKCTVLDLPKVIDKVPTDGVITYVAGDLFHSIPSSQAMMLKLVLHFWSDEDCVRILTHCRKAIPPRDEGGKLIIIDIVVGPSLGPVMFEAQLLMDMLMMVNTRGGQRDENDWRKIFIKAGFTDYKIMKKLGARCVIEVYP

>AsCOMT34 Accession ID: XP_044443717.1

MGSTAADMAASADEEACMFALQLASSSILPMTLKNAIELGLLETLVAVSGKSLTPSEVAAKLPSAANPAAPDMVDRMLRLLASYNIVSCVVEEGKDGRLSRRYGAAPVCKYLTPNEDGVSMAALALMNQDKVLMESWYYLKDAVLDGGIPFNKAYGMSAFEYHGTDPRFNRVFNEGMKNHSIIITKKLLELYDGFKGLGTLVDVGGGVGATVAAITAHYPTIKGINFDLPHVISEAPPFPGVTHVGGDMFKKVPSGDAILMKWILHDWSDQHCATLLKNCYDALPAHGKVVLVECILPVNPEAKPSSQGVFHVDMIMLAHNPGGRERYEREFEALARGAGFAGVKSTYIYANAWAIEFTK

>AsCOMT37 Accession ID: XP_044443717.1

MATSSNEELLQAHAELWSLTFSYLKSMALECAIKLGTPNAIHRRGGAVSLPDLLAAIPVPESKKPYLPRLMRFLVASGIFAADADGSAAPTYRLTPLSRLLVDDGTAAAAGAADLRSVDLSPFVLSQTNRYHVTAATHLSEWFESDAAAEMPFRTAHGADPWMVFAHDPKINMVFNAGMAADTRFAMSFVLSNYGDAFDGVTSLVDVAGGTGTAARAIANAFPRIKCSVLDLPNVIDSVPADGVVEYVAGDMMSSIPKADAMFLKYVLHDWNDEDCVRILRECRKAILKPGGKVIIVDMVVGSPSNNAAYEAQVLFDLLMMVITAGKERDEREWTKIFMDAGFSHHKTRPVLGSVALIELYP

>AsCOMT36 Accession ID: AAK68909.1

MAAQAQLTVPTDAELLQAQADLWRHSLYYMTSMAFQCAVKLGIPTTIHGLGGAASLPQLVTALSLPPAKLPYLRRIMRLLATSGVFATADVDVYRLTPISFLLLDGVDIDGHPSQTSVVLAATSRHCVEAALGLTDWFKKDVAGSPFEDLHGVTLFDGSMAEADPEIDAVFNEGLAAHDNSGFLAVLRECGDTLFQGLDSLTDCCGGDGTTARSIVEAFPQIKCTVLDLPRVIDNIPTDGVVNYVAGDMFKLVPPAQAVLVKLVLHHWSDEDCVKILAQCKKAIPSREEGGKVIVIDILVDPSSGPMHEAELLVDVAMMVFTNGRQRDESDWGEIFTKAGFTGYNIVKKLGARGVFEVYP

>AsCOMT35 Accession ID: XP_047091613.1

MAPQVPTIEVPTDAELLQAQADLWRHSLCYLTSMGLRCAVKLGIPTAIHNLGGVSSLPDLITALSIPASKQPFLGRLMRGLVTSGVFAAGNNSEAELFRLNPLSRTLVDGVVADDHHIQTSLVLVTTSPHYLEAALGLADWFMKDVVGPVPSPFEDVHGASLFDESTALLDKDFDAVANAALAAHDNQGMGTVLRQCHDLFKGLESLTDCGGGDGATARAITKAHPHVKCTVLDLPKVIDKVPSDGIVNYVAGDLFHSVPKSQAVMLKLVLHHWSDEDCVKILSQCRSAIPSREEGGKVIIIDILVGPSLGPIMFEAQVLMDMAMLVNTRGRQRDENDWRQLFMKAGFSDYKIVKKVGARGVFEVYK

>AsCOMT21 Accession ID: XP_051178062.1

MGSAAELFVVPAVANDEEACTYAIELAAACILPMTLKSTIELGLLEILVGAGGKALSPSEVVARLPSGTSNPDAPAMVDRMLRLLASFNVVSCQVEEGTDGLLARRYSPAPVCKWLTPNEDGVSMAPLVLARNDKVTMESWYHLTSAVLDGGLPFDKAHGMPAFKYYGTDARFNRLFNEAMKNHSTIITKKLLDSYTGFDDVGTLVDVGGGTGATIRAITSKYPHIKGINLDLPHVISEALPSPGVHHVCGDMFTEVPSGDAILMKWILHDWADENCLTLLRKCYDALPTHGKVILVEFILPENMNDTTKGVFMADMAMLTNTPGGKERYRREFEVLANCAGFGSFKATYLYANVWVIEFTK

>AsCOMT31 Accession ID: XP_047080235.1

MPSPAAEPTAAVAELSPAEARLAMMELANMISVPMALTAVIRLGVPPAIWAGGANAPLPAAALLPAGHPDPSVLERLLRLLTSHSVFSEHAATDGGGGTRRYALTAVGRTLVPATGASSGASYADYVLQHHQDELVHAWPRLRDAVLDPAGPEAFARVHNGVSAYAYYGQEATELMMGAMTGVSKPFMEMLLDGYVGGFEGVETLVDVGGSSCLCLEMIMRRVTTIKEGVNFDLPSVVAAAPPIDGVRDALELSPLYFPRSR

*Arabidopsis thaliana (L.)*

>AT1G21100.1 Accession ID: NP_173534.1

MGYLFQETLSSNPKTPIVVDDDNELGLMAVRLANAAAFPMVLKAALELGVFDTLYAAASRTDSFLSPYEIASKLPTTPRNPEAPVLLDRMLRLLASYSMVKCGKALSGKGERVYRAEPICRFFLKDNIQDIGSLASQVIVNFDSVFLNTWAQLKDVVLEGGDAFGRAHGGMKLFDYMGTDERFSKLFNQTGFTIAVVKKALEVYEGFKGVKVLVDVGGGVGNTLGVVTSKYPNIKGINFDLTCALAQAPSYPGVEHVAGDMFVDVPTGDAMILKRILHDWTDEDCVKILKNCWKSLPENGKVVVIELVTPDEAENGDINANIAFDMDMLMFTQCSGGKERSRAEFEALAAASGFTHCKFVCQAYHCWIIEFCK

>AT1G21110.1 Accession ID: NP_173535.1

MGYLFEETLSSNPKTPIVVDDDNELGLMAVRLANAAAFPMVLKASLELGVFDTLYAEASRTDSFLSPSEIASKLPTTPRNPGAPVLLDRMLRLLASYSMVKCEKVSVGKEQRVYRAEPICRFFLKNNIQDIGSLASQVIVNFDSVFLNTWAQLKDVVLEGGDAFGRAHGGMKLFDYMGTDERFSKLFNQTGFTIAVVKKALEVYQGFKGVNVLVDVGGGVGNTLGVVTSKYPNIKGINFDLTCALAQAPTYPGVEHVAGDMFVDVPTGNAMILKRILHDWTDEDCVKILKNCWKSLPQNGKVVVIELVTPDEAENGDINANIAFDMDMLMFTQCSGGKERSRAEFEALAAASGFSHCQFVCQAYHCWIIEFCK

>AT1G21120.2 Accession ID: NP_173536.2

MHQKYTNSSSQHYKKETNTLSRYIFPHFDQFDHVECLEGEESNKKEREMGYLFEETLSSNPKTPIVVDDDNELGLMAVRLANAAAFPMVLKASLELGVFDTLYAEASRTDSFLSPSEIASKLPTTPRNPGAPVLLDRMLRLLASYSMVKCEKVSVGKGERVYRAEPICRFFLKNNIQDIGSLASQVIVNFDSVFLNTWAQLKDVVLEGGDAFGRAHGGMKLFDYMGTDERFSKLFNQTGFTIAVVKKALEVYQGFKGVNVLVDVGGGVGNTLGVVTSKYPNIKGINFDLTCALAQAPSYPGVEHVAGDMFVDVPTGDAMILKRILHDWTDEDCVKILKNCWKSLPENGKVVVIELVTPDEAENGDINANIAFDMDMLMFTQCSGGKERSRAEFEALAAASCFTHCKFVCQAYHCWIIEFCK

>AT1G21130.1 Accession ID: NP_173537.1

MGYLLEETLSSNSKTPIVIDDDNELGLMAVRLANAAAFPMVLKAALELGVFDTLYAEASRSDSFLSPSEIASKLPTTPRNPEAPVLLDRMLRLLASYSVVKCGKVSEGKGERVYRAEPICRFFLKDNIQDIGSLASQVIVNFDSVFLNTWAQLKDVVLEGGDAFGRAHGGMKLFDYMGTDERFSKLFNQTGFTIAVVKKALEVYQGFKGVNVLVDVGGGVGNTLGVVASKYPNIKGINFDLTCALAQAPSYPGVEHVAGDMFVDVPTGDAMILKRILHDWTDEDCVKILKNCWKSLPESGKVVVIELVTPDEAENGDINANIAFDMDMLMFTQCSGGKERSRAEFEALAAASGFTHCKFVCQAYHCWIIEFCK

>AT1G33030.1 Accession ID: NP_175611.1

MEEQNLSSYAMILSSSSVLPMVLKTAIDLGLFDILAESGPSSASQIFSLLSNETKKHHDSSLVNRILRFLASYSILTCSVSTEHGEPFAIYGLAPVAKYFTKNQNGGGSLAPMVNLFQDKVVTDMWYNLKDSVLEGGLPFNNTHGSSAVELVGSDSRFREVFQSSMKGFNEVFIEEFLKNYNGFDGVKSLVDVGGGDGSLLSRIISKHTHIIKAINFDLPTVINTSLPSPGIEHVAGDMFTNTPKGEAIFMKWMLHSWDDDHCVKILSNCYQSLPSNGKVIVVDMVIPEFPGDTLLDRSLFQFELFMMNMNPSGKERTKKEFEILARLAGFSNVQVPFTSLCFSVLEFHKNK

>AT1G51990.1 Accession ID: NP_174579.1

MISLQTSGGSSEEEDMLLAIQLGGLNFVPYIVKTARELDLFEIMAKARPLGSYLSPVDLASMAAPKNPHAPMMIDRLLRFLVAYSVCTCKLVKDEEGRESRAYGLGKVGKKLIKDEDGFSIAPYVLAGCTKAKGGVWSYLTEAIQEGGASAWERANEALIFEYMKKNENLKKIFNESMTNHTSIVMKKILENYIGFEGVSDFVDVGGSLGSNLAQILSKYPHIKGINFDLPHIVKEAPQIHGVEHIGGDMFDEIPRGEVILMKWILHDWNDEKCVEILKNCKKALPETGRIIVIEMIVPREVSETDLATKNSLSADLTMMSLTSGGKERTKKEFEDLAKEAGFKLPKIIYGAYSYWIIELYPN

>AT1G63140.4 Accession ID: NP_001321812.1

MNTIYTFITYLSRFSNQTTTMENHLQHSLTIIPKPDLIKEEQRYHEDTVSLQAERILHAMTFPMVLKTALELGVIDMITSVDDGVWLSPSEIALGLPTKPTNPEAPVLLDRMLVLLASHSILKYRTVETGDNIGSRKTERVYAAEPVCTFFLNRGDGLGSLATLFMVLQGEVCMKPWEHLKDMILEGKDAFTSAHGMRFFELIGSNEQFAEMFNRAMSEASTLIMKKVLEVYKGFEDVNTLVDVGGGIGTIIGQVTSKYPHIKGINFDLASVLAHAPFNKGVEHVSGDMFKEIPKGDAIFMKWILHDWTDEDCVKILKNYWKSLPEKGKVIIVEVVTPEEPKINDISSNIVFGMDMLMLAVSSGGKERSLSQFETLASDSGFLRCEIICHAFSYSVIELHK

>AT1G76790.1 Accession ID: NP_177805.1

MGHLIPQTGDEETELGLAAVRLANCAAFPMVFKAAIELGVIDTLYLAARDDVTGSSSFLTPSEIAIRLPTKPSNPEAPALLDRILRLLASYSMVKCQIIDGNRVYKAEPICRYFLKDNVDEELGTLASQLIVTLDTVFLNTWGELKNVVLEGGVAFGRANGGLKLFDYISKDERLSKLFNRTGFSVAVLKKILQVYSGFEGVNVLVDVGGGVGDTLGFVTSKYPNIKGINFDLTCALTQAPSYPNVEHVAGDMFVDVPKGDAILLKRILHDWTDEDCEKILKNCWKALPENGKVIVMEVVTPDEADNRDVISNIAFDMDLLMLTQLSGGKERSRAEYVAMAANSGFPRCNFVCSAYHLWVIELTKQA

>AT1G77520.1 Accession ID: NP_177876.1

MTNHLQDPLPTYPKPVLTKEEQEVDEKMVSLQAESIVNTVAFPMVLKAAFELGVIDTIAAAGNDTWLSPCEIACSLPTKPTNPEAPVLLDRMLSLLVSHSILKCRMIETGENGRTGKIERVYAAEPVCKYFLRDSDGTGSLVPLFMLLHTQVFFKTWTNLKDVILEGRDAFNSAHGMKIFEYINSDQPFAELFNRAMSEPSTMIMKKVLDVYRGFEDVNTLVDVGGGNGTVLGLVTSKYPHIKGVNFDLAQVLTQAPFYPGVEHVSGDMFVEVPKGDAVFMKWILHDWGDEDCIKILKNCWKSLPEKGKIIIVEFVTPKEPKGGDLSSNTVFAMDLLMLTQCSGGKERSLSQFENLAFASGFLRCEIICLAYSYSVIEFHK

>AT1G77530.1 Accession ID: NP_177877.1

MSNHLQDPLTTYPKPGLTKEEQEIDEKMVSLQAESIVNAVAFPMVLKAALELGVIDTIAAASNGTWLSPSEIAVSLPNKPTNPEAPVLLDRMLRLLVSHSILKCCMVESRENGQTGKIERVYAAEPICKYFLKDSDGSGSLSSLLLLLHSQVILKTWTNLKDVILEGKDAFSSAHDMRLFEYISSDDQFSKLFHRAMSESSTMVMKKVLEEYRGFEDVNTLVDVGGGIGTILGLITSKYPHIKGVNFDLAQVLTQAPFYPGVKHVSGDMFIEVPKGDAIFMKWILHDWGDEDCIKILKNCWKSLPEKGKVIIVEMITPMEPKPNDFSCNTVLGMDLLMLTQCSGGKERSLSQFENLAFASGFLLCEIICLSYSYSVIEFHK

>AT3G53140.1 Accession ID: NP_190882.1

MENESSESRNRARLAIMELANMISVPMSLNAAVRLGIADAIWNGGANSPLSAAEILPRLHLPSHTTIGGDPENLQRILRMLTSYGVFSEHLVGSIERKYSLTDVGKTLVTDSGGLSYAAYVLQHHQEALMRAWPLVHTAVVEPETEPYVKANGEAAYAQYGKSEEMNGLMQKAMSGVSVPFMKAILDGYDGFKSVDILVDVGGSAGDCLRMILQQFPNVREGINFDLPEVVAKAPNIPGVTHVGGDMFQSVPSADAIFMKWVLTTWTDEECKQIMKNCYNALPVGGKLIACEPVLPKETDESHRTRALLEGDIFVMTIYRTKGKHRTEEEFIELGLSAGFPTFRPFYIDYFYTILEFQK

>AT4G35150.1 Accession ID: NP_195241.1

MEESKRNLLDEEAKASLDIWRYVFGFADIAAAKCAIDLKIPEAIENHPSSQPVTLSELSSAVSASPSHLRRIMRFLVHQGLFKEVPTKDGLATGYTNTPLSRRMMITKLHGKDLWAFAQDNLCHSQLINEAMACDARRVVPRVAGACQGLFDGVATVVDVGGGTGETMGILVKEFPWIKGFNFDLPHVIEVAQVLDGVENVEGDMFDSIPASDAVIIKWVLHDWGDKDCIKILKNCKEAVLPNIGKVLIVECVIGEKKNTMIAEERDDKLEHVRLQLDMVMMVHTSTGKERTLKEWDFVLTEAGFARYEVRDFDDVQSLIIAYRS

>AT4G35160.1 Accession ID: NP_195242.1

MSSDQLSKFLDRNKMEDNKRKVLDEEAKASLDIWKYVFGFADIAAAKCAIDLKIPEAIENHPSSQPVTLAELSSAVSASPSHLRRIMRFLVHQGIFKEIPTKDGLATGYVNTPLSRRLMITRRDGKSLAPFVLFETTPEMLAPWLRLSSVVSSPVNGSTPPPFDAVHGKDVWSFAQDNPFLSDMINEAMACDARRVVPRVAGACHGLFDGVTTMVDVGGGTGETMGMLVKEFPWIKGFNFDLPHVIEVAEVLDGVENVEGDMFDSIPACDAIFIKWVLHDWGDKDCIKILKNCKEAVPPNIGKVLIVESVIGENKKTMIVDERDEKLEHVRLMLDMVMMAHTSTGKERTLKEWDFVLKEAGFARYEVRDIDDVQSLIIAYRS

>AT5G37170.1 Accession ID: NP_198533.1

MTNHHQESLTTYPKPGPTREQEQVDEEMMSMQMQALRITNSLAFPMGVWLSPSEIAFGLPTKPTNPEAPMLIDRMLRLLVSHSILKCRLVETGENNRTESTQRVYAAEPDTSEGCDTRRKRCIQFCPWHGTLRIRCTDEQFAAIFNQAMSDSSTMIMTKILEVYKGLKDVNTLVDIGGGLGTILNLVISSKYPQIKGINFDLAAVLATAPSYPGVEHVPGDMFIDVPKGDAIFMRRILRDWNDKDCVKILTNCWKSLPEKGKVIIVDMVAPSEPKSDDIFSKVVFGTDMLMLTQCSCGKVRSFAQFEALASASGFHKCEVSGLAYTYSVIEFHK

>AT5G53810.1 Accession ID: NP_200192.1

MANHLQVPLTKPDRVKEEQEVEEEARLLARRLANAAASPMVLKAALELGVIDTITTVGGGDLWLSPSEIALRLPTKPCNLEAPALLDRMLRFLVSHSVLKCRTVIEENGQTGKVERVYAAEPVCKYLLNKSDDVSGSFASLFMLDLSDVFIKTWTHLEDVILEGRDAFSSAHGMKLFEYIQADERFGKVFNRAMLESSTMVTEKVLKFYEGFKDVKTLVDVGGGLGNTLGLITSKYPHLIGINFDLAPVLANAHSYPGVNHVAGDMFIKIPKGDAIFMKWILHDWTDEQCVAILKNCWKSLEENGKLIIVEMVTPVEAKSGDICSNIVFGMDMTMLTQCSGGKERDLYEFENLAYASGFSRCAIVCAVYPFSVIEIYK

>AT5G54160.1 Accession ID: NP_200227.1

MGSTAETQLTPVQVTDDEAALFAMQLASASVLPMALKSALELDLLEIMAKNGSPMSPTEIASKLPTKNPEAPVMLDRILRLLTSYSVLTCSNRKLSGDGVERIYGLGPVCKYLTKNEDGVSIAALCLMNQDKVLMESWYHLKDAILDGGIPFNKAYGMSAFEYHGTDPRFNKVFNNGMSNHSTITMKKILETYKGFEGLTSLVDVGGGIGATLKMIVSKYPNLKGINFDLPHVIEDAPSHPGIEHVGGDMFVSVPKGDAIFMKWICHDWSDEHCVKFLKNCYESLPEDGKVILAECILPETPDSSLSTKQVVHVDCIMLAHNPGGKERTEKEFEALAKASGFKGIKVVCDAFGVNLIELLKKL

*Zea mays L.* Accession ID:

>Zm00001eb018630 Accession ID: XP_020402727.1

MLQAHDELLHHSLCFAKSLALTVALDLRIPDAIHHHGGGATLLQIIAETGLHPSKLRALGRLMRVLTVTGTFSVQQPPAGSDDDEAVVVYRLTAASRFLVSDEVSTATTLAPFVSLALQPIAASPHALGICAWFRQEQHEPSPYGLAFRQTPTLWEHADDVNALLNKGMVADSRFLMPIVLRQCGEMFRGINSLVDVGGGHGGAAAAIAAAFPPLQEKPKISAHVKCSVLDLPHVVAGAPSDGNVHFVAGNMFESIPPATAVFLKKTLHDWGDDECVKILKNCKQAIPPRDAGGKVIILDVVVGYKQSNIKHQETQVMFDLYMMAVNGVERDEQEWKKIFAEAGFKDYKILPVIGDVSVIIEVYP

>Zm00001eb018640 Accession ID: NP_001140761.1

MALIMQESSSHDLLQAHDELLHHSLCFAKSLALAVALDLRIPDAIHHHGAGGATLLQILAETALHPSKLRALRRLMRVLTVTGIFSVEQPPAGGGDNSTVHTSDDEAVVVYRLTAASRFLVSDEVSTATLAPFVSLALQPIAACPHALGISAWFRQEQHEPSPYGLAFRQTPTIWEHADDVNALLNKGMAADSRFLMPIVLRECGETFRGIDSLVDVGGGHGGAAAAIAAAFPHLKCSVLDLPHVVAGAPSDGNVHFVAGNMFESIPPATAVFLKKTLHDWGDDECVKILKNCKQAISPRDAGGKVIILDVVVGYKQSNIKHQETQVMFDLYMMAVNGVERDEQEWKKIFIEAGFKDYKILPVIGDVSVIIEVYP

>Zm00001eb018660 Accession ID: NP_001306664.1

MALMQESSSQDLLQAHDELLHHSLCFAKSLALAVALDLRIPDAIHHHGAGGATLLQILAETALHPSKLRALRRLMRVLTVTGIFSVVEQPPAGGGDDSTVHTSDDEAVVVYRLTAASRFLVSDDVSTATLAPFVSLALQPIAACPHALGISAWFRQEQHEPSPYGLAFRQTPTIWEHADDVNALLNKGMAADSRFLMPIVLRECGETFRGIDSLVDVGGGHGGAAATIAAAFPHLKCSVLDLPHVVAGAPSDGNVQFVAGNMFESIPPATAVFLKKTLHDWGDDECVKILKNCKQAISPRDAGGKVIILDVVVGYKQSNIKHQETQVMFDLYMMAVNGVERDEQEWKKIFTEAGFKDYKILPVIGDVSVIIEVYP

>Zm00001eb040570 Accession ID: NP_001146259.1

MSSVQEELNNTQDMLQGYVELYNYSLSYVKTMAIGCAIQLGIPSAIHRRGGAATISDIITETGVDPSKLPYLRRLMRVLTVSSILATTGTDETETESDDSTVYKLTPASRLLVSGAGAPTSCDISPMLDLLMRPTTSVATYFSLEEWFKDAGATATLFEVAHGMSPWSLTKNDALYNKTLNDGCAADSNFAMDTLLREPRAAGIFRGLGSLVDVGGGHGAAAMAIARAFPHIRCSVLDLEQVVSGAPDDGTVKFIAGDMFESIPAADCVLLKYVLHCWDDESSVKILRQCKRAIPARDAGGKVVIMNMVVGYGSSDRFVKETQVMCDMWMMRYVGVEREEHEWKRIFLEAGFSDYRITPTALGFQSVIEVFP

>Zm00001eb090230 Accession ID: NP_001132142.1

MAALAPSIVVPTDAELLQAQADLWRNSLCYLKSMALKCATELGIPTAIYSLGGAASLPDLIASLSLPQAKLPFLGRLMRLLSSSGVFAVVESPEAVYSLTPLSYLLVDGIAADNNHMDHAPFLLTVTSAHYIDLAIDLADWFKKEAKTPPFDHKHAASLFEESMERKAPGFHKMSILGLLVHDNFATSIAVREYQDVFQGVKSVTDCCYHGDGTTGKALAKAFPLIKITVLDLPQEIRKIPADGVVNYVGGDMFKSIPRAQMVLLKMVLHHWSDEDCVKILANCRKAIPSREEGGKVVIADIILDPASGPVMFQTQLLMDVCMMLMKGGRQRDVNDWRDLIQKAGFSDYKLLKKFGARGVLEIYP

>Zm00001eb090830 Accession ID: NP_001106076.1

MASEVVRPSDAELLKAQADIWRLSLSYLTPLSLRCAVELGIPTAIYRHGGAASAAELVTALSLPSTKLPFLRRLLRLLAASGVFTVDKQSSEEERYRISPVSYLLVDGIPHEDHMNHTALVLTCTSTRYIEAGIGLAEWFKRDVVTSPFEELHGATLFHESMGSLDADFHDMASEALDAHDNFGIEIAMREFRDLFEGIQSMTYCCGNFGDDKGARAIVKAFPHIKCTVLAPPKIIATKPADGAMINYVEGDMFSFIPPAQTVVLKVM

>Zm00001eb092770 Accession ID: NP_001147709.1

MALMQESSQDLLEAHDELFHHCLCFAKSLALAVAQDLRIPDAIHHHGGGATLHQILAEAALHPSKLRALRRLMRVLTVSGVFTVQYSSTVDASDGADVVYRLTAASRFLVSDSDEAGTASLAPFANLALHPIAISPHAVGICAWFRQEQHDPSPYGLAFRQIPTIWEHADNVNALLNKGLLAESRFLMPIVLRECGDEVFRGIDSLVDVGGGHGGAAATIAAAFPHVKCSVLDLPHVVAGAPSDACVQFVAGNMFHSIPPATAVFFKVPSNPLHALHAADRLPDNSID

>Zm00001eb164750 Accession ID: NP_001150654.1

MATAIVPTDAELLQAQADLWRHSLYYLTSMALKCAVELHIPTAIHNLGGSATLPDLVAALSLPAAKLPFLGRVMRLLVTSGVFASSDDVQYRLNPLSWLLVEGVESEDHTYQKYFVLGTVSRHYVEAGMSLADWFKKEEDEDRQLPSPFEALHGVPLVHESTKLLDEELDRVVEEGVAAHDNLAIGTVIRECGADVFSGLRSLTYCCGRQGNASAAAIVKAFPDIKCTVLNLPRVVEETTTKTITIPPAQAVMLKLVLHFWSDDDCVKILELCRKAIPSRQEGGKVIIIEILLGPYMGPVMYEAQLLMDMLMMVNTKGRQRGEDDWRHIFTKAGFSDYKVVKKIGARGVIEVYP

>Zm00001eb169520 Accession ID: NP_001168311.2

MGHQAQHGTDDTEELLAAHRQLWCHALGYVKSMALKCALDLRIPDTIDRCGGSATLGELLAASEIPASNHDYLRRVMRTLTAMRIFAVSHDDPAKADDAAAISYQLTPASRLLVSSSSSSVDAAAAGAGASKENTTTPSILPNIAHLVRPNTISLLFSMGEWMKDESAASVSLYETVHRQGMWACVEDDAANRASFYESMDADTRLVMQAVVRRCPHVFDGIKSLVDVGGGRGTAAAAVVAAFPHIQRCTVMDLPHVVAEAPAGTAGLSFHGGDMFEHIPSADALMLKWILHDWDEDKCIKIMERCKEAIGGKEAGGKVIIIDTVLGSRADDDDDDKTCRETYVLDLHILSFVNGAEREEHEWRRIFLAAGFRDYKITHTRGIPSIIEVFP

>Zm00001eb170590 Accession ID: NP_001168311.2

MAPIKEQKHTTSAEQQVMLDAELQLWNHTFGYVKSMALKAAIDLGIPEAIHQHGGTATLPQIVTRVKLHPSKTPCLRRLMRVLTLTGVFGAQEPHDDDGGCDDELVYTLTPASRLLVGSPGQNVGPFLTLMLGPIFVSSFLDLRGWFQHETPDPSPFKMTHGRDIWELAAHDAAFGRLFDAGMVADSSFIMDVVVRECGGVFEGISSLVDVAGGLGGATQTIAKAFPNLECSVLDLPNVVASAPADTAVKYVPGDMFESVPAADAVFLKILKNCKKAIPAQGGKVIILDIVVGAGSSCDRKNVETQCLFDLFIMFINGAERDERQWKKIIFEAGFTSYKIIPVLGIRSIIEICL

>ZmNP_001105689.1 Accession ID: NP_001105689.1

MELSPNNSTDQSLLDAQLELWHTTFAFMKSMALKSAIHLRIADAIHLHGGAASLSQILSKVHLHPSRVSSLRRLMRVLTTTNVFGTQPLGGGSDDDSEPVYTLTPVSRLLIGSQSSQLAQTPLAAMVLDPTIVSPFSELGAWFQHELPDPCIFKHTHGRGIWELTKDDATFDALVNDGLASDSQLIVDVAIKQSAEVFQGISSLVDVGGGIGAAAQAISKAFPHVKCSVLDLAHVVAKAPTHTDVQFIAGDMFESIPPADAVLLKSVLHDWDHDDCVKILKNCKKAIPPREAGGKVIIINMVVGAGPSDMKHKEMQAIFDVYIMFINGMERDEQEWSKIFSEAGYSDYRIIPVLGVRSIIEVYP

>ZmNP_001146259.1 Accession ID: NP_001146259.1

MSSVQEELNNTQDMLQGYVELYNYSLSYVKTMAIGCAIQLGIPSAIHRRGGAATISDIITETGVDPSKLPYLRRLMRVLTVSSILATTGTDETETESDDSTVYKLTPASRLLVSGAGAPTSCDISPMLDLLMRPTTSVATYFSLEEWFKDAGATATLFEVAHGMSPWSLTKNDALYNKTLNDGCAADSNFAMDTLLREPRAAGIFRGLGSLVDVGGGHGAAAMAIARAFPHIRCSVLDLEQVVSGAPDDGTVKFIAGDMFESIPAADCVLLKYVLHCWDDESSVKILRQCKRAIPARDAGGKVVIMNMVVGYGSSDRFVKETQVMCDMWMMRYVGVEREEHEWKRIFLEAGFSDYRITPTALGFQSVIEVFP

>ZmNP_001105914.1 Accession ID: NP_001105914.1

MAHAALLHCSQSSRSLAACRRGSHYRAPSHVPRHSRRLRRAVVSLRPMASSTAQAPATAPPGLKEGIAGLYDESSGLWENIWGDHMHHGFYDSSEAASMADHRRAQIRMIEEALAFAGVPASDDPEKTPKTIVDVGCGIGGSSRYLAKKYGAQCTGITLSPVQAERGNALAAAQGLSDQVTLQVADALEQPFPDGQFDLVWSMESGEHMPDKRKFVSELARVAAPGGTIIIVTWCHRNLDPSETSLKPDELSLLRRICDAYYLPDWCSPSDYVNIAKSLSLEDIKTADWSENVAPFWPAVIKSALTWKGFTSLLTTGWKTIRGAMVMPLMIQGYKKGLIKFTIITCRKPGAA

>ZmNP_001106047.1 Accession ID: NP_001106047.1

MGSTAGDVAAVVDEEACMYAMQLASSSILPMTLKNAIELGLLEVLQKEAGGGKAALAPEEVVARMPAAPGDPAAAAAMVDRMLRLLASYDVVRCQMEDRDGRYERRYSAAPVCKWLTPNEDGVSMAALALMNQDKVLMESWYYLKDAVLDGGIPFNKAYGMTAFEYHGTDSRFNRVFNEGMKNHSVIITKKLLDFYTGFEGVSTLVDVGGGVGATLHAITSRHPHISGVNFDLPHVISEAPPFPGVRHVGGDMFASVPAGDAILMKWILHDWSDAHCATLLKNCYDALPENGKVIVVECVLPVNTEATPKAQGVFHVDMIMLAHNPGGKERYEREFRELAKGAGFSGFKATYIYANAWAIEFIK

>ZmNP_001131705.1 Accession ID: NP_001131705.1

MFTRAELLEASVELRHHALGCVKSTALRCAVKLGVANAIQRRGGRASVEDLLTELSLDASRLRCLHSVMRALAALGVFKEGSDGEYGLTAISSLLVDDDSSVRGSLRPITLLYLEPAFVAPVLNLADWALAGVDGSDDDNNCSARGTAFKMTHGEDVWDVLARDAFLGDFFNGALASETRFLMDIAIRGSPQVFEGIASLVDAGGGTGAAAQAVAAAFPDTRCTVLELPQVVDAAPIDGPVRFVGGDMTKFIPPADAVLLKNVLHDWSDKDCVIILKRCKEAIAASGKVIVIDIVLGSSSLAICNETQLWLDLFMSTVTTGKERREEEWYRLFKEAGFSAYKISPVLGLLSIIEVFL

>ZmNP_001132142.1 Accession ID: NP_001132142.1

MAALAPSIVVPTDAELLQAQADLWRNSLCYLKSMALKCATELGIPTAIYSLGGAASLPDLIASLSLPQAKLPFLGRLMRLLSSSGVFAVVESPEAVYSLTPLSYLLVDGIAADNNHMDHAPFLLTVTSAHYIDLAIDLADWFKKEAKTPPFDHKHAASLFEESMERKAPGFHKMSILGLLVHDNFATSIAVREYQDVFQGVKSVTDCCYHGDGTTGKALAKAFPLIKITVLDLPQEIRKIPADGVVNYVGGDMFKSIPRAQMVLLKMVLHHWSDEDCVKILANCRKAIPSREEGGKVVIADIILDPASGPVMFQTQLLMDVCMMLMKGGRQRDVNDWRDLIQKAGFSDYKLLKKFGARGVLEIYP

>ZmNP_001140567.1 Accession ID: NP_001140567.1

MEASSRLELQQAHTELWNLTFSYLKSMALECVVQLNIPNVIHNFGGNATLPSILSAIQVPEHRKPYLPRLMRFLVVSGILSFDSPTLGEEGTYHLTPLSRLLVDDTHINGYGSLGPFVLSQTTKYHVSSATYLSEWFKGEDGAGPMAAEMPFKMAHGTGPWGALGHDQQFNRVFNAGLGSNSRLVLDFVVAEYGDVFDGVSSLLDVGGGDGSTARTIRKAFPHIKCSVLDLPNVIIDIQPGDGMVDYIAGDMFSSIPPTDAIMLKYVLHDWNDDDCVKILQQCKKAICSCKPAGGKVLIIDVVVGSPLKEMFEAQVTSDLLMMVIAGGKERDKKEWHKIFVESGFKDYKISPVLGYLSIIELYS

>ZmNP_001146259.1 Accession ID: NP_001146259.1

MSSVQEELNNTQDMLQGYVELYNYSLSYVKTMAIGCAIQLGIPSAIHRRGGAATISDIITETGVDPSKLPYLRRLMRVLTVSSILATTGTDETETESDDSTVYKLTPASRLLVSGAGAPTSCDISPMLDLLMRPTTSVATYFSLEEWFKDAGATATLFEVAHGMSPWSLTKNDALYNKTLNDGCAADSNFAMDTLLREPRAAGIFRGLGSLVDVGGGHGAAAMAIARAFPHIRCSVLDLEQVVSGAPDDGTVKFIAGDMFESIPAADCVLLKYVLHCWDDESSVKILRQCKRAIPARDAGGKVVIMNMVVGYGSSDRFVKETQVMCDMWMMRYVGVEREEHEWKRIFLEAGFSDYRITPTALGFQSVIEVFP

*Oryza sativa*

>LOC_Os04g43940.1 Accession ID: BAD38255.1

MTNQWREAILGITMDGGQNRRCLNTNSEAGDEATEDPYAYYPGFPSYRVRQCGSVWEARVEIHPRVPSELDYSFQTRHHRDLPETAMQDAAREAFLRLSSIHRAELVSTEFAHHPFREEGNSVCTVQDTPCCYNPMVTRLSRWAEAVDDYYEEALLEIDDHQRRVGELETEVTDLTAQQLQLEEEHQAWGSEIDSLKAQLQVHGDQFQQLSDQYNDRRGMMDTLEEQLRESQEHVCQLEEQLRAAKISTSGASTSTAVGRDRYFFLTPPYPPAFHALLGEASMLATESAPYLVDPTTQPPVPELVHTPLIPTPSPQLGSSLETPIQVDSETEGTDTEPEIEPDITDPSEDEAPVPRITFLGGPRTLSTARKSTRPPGKRPKPDPEATTSEPWGLRFARASDHPLPAPGSCGWLED

>LOC_Os09g13280.1 Accession ID: AAN11185.1

MDRSWIYATTWKLHCKEYRDGILSFMNAAEEDRKRRNNKYMCCQGRSNMFKGDCKNKNMFESRVDDLHSHLIQRGFMKGYTCWAKHGEQELGSGAVADRSGADNDEEGDEDEHDMFIPSPLDGEMIDVDLDLLQDMLRDVEDPSYNEKDSMKFSRLVSDSEIAL

>LOC_Os08g06850.1 Accession ID:ABB47865.2

MQCFYVSRGKRSEPPLSPEEEAKFEASDCLFRGALISVLADNIVDVYMHMPSGKDMWDALEAKFGVSDAGSELYVMEQFYDYKMVDDRSVVEQAHEIQMLAKELENNNCELPDKFVAGGIIAKLPPSWSDFATSLKHKRQEFSVPDLIGSLGVEEKARAKDVRGKKVEGGSSANMVQKKNPHASHNNKKVKPDVKPKAATNFKKKSKGKAKGDCFVCGKSGHWAKDCPERKDRKSANMIISEGGGTSRYGKILPTVLSVFHSPDWWVDTGANIHVGRGSSLLMGNGSLAAVHGVGTVDLKFTSGKTVQLKNVQHVPSIKKNLVSGSLLCREGFRLVFESNKCVVSKYGTFVGKGYDSGGLFRFSLNDMCNNHNAVNHISENDESNDEALHFFKIYKAEVENQLERKIKWLRSDRGGEYFSNEFVSFCEEFGIIHEMTPPYSPQSNGVAERKNRTLTEMVNAMLDTAGLSKDGGHKEVTPFEEWERKKLNLSYLRTWGCLAKVNVPIAKKRKLGPKTVDCVFLGYAIHSVGYRFLIVNSGVPDMHVGTILESRDATFFENEFPMKYTPSTSSKETVMPHEHFAPIEHNDQTPEENPEEDNIVDTRKSKRQRVAKSFGDDYIVYLVDDTPRTIEEAYSSPDADYWKEAVRSEMDSIMSNGTWEVVERPYGCKPVGCKWVFKKKLRPDGTIEKYKARLVAKGYTQKEGEDFFDTYSPVARLTTIRVLLALAASHSLLVHQMDVKTAFLNGELEEEIYMDQPDGYVLEGQEGMVCKLLKSLYGLKQAPKQWHEKFDTTLTSVGFVVNEADKCVYYRYGGGEGVILCLYVDDILIFGTSLNMIEEVKDYLSKSFEMKDLGEADVILNIKLQRGDEGGITLVQSHYVDKVLSRFGYSDCKPAPTPYDPSVLLRKNRRIARDQLRYSQIIGSLMYLASATRPDISFAVSKLSRFVSNPGDDHWQALERVMRYLKGTMSYGIHYTGYPKVLEGYSDSNWISDADEIKATSGYVFTLGGGAVSWKSCKQTILTRSTMEAELTALDTATVEAEWLRELLMDLPVVEKPVPAILMNCDNQTVIIKVNSSKDNMKSSRHIKRRLKSVRKQKNSGVIALDYVQTARNLADQFTKGLPRNVIDSASREMGLIPT

>LOC_Os02g44050.1 Accession ID: ABA91263.1

MAEPAKAHDLSPSMSGDDGEPNPRRRARTPPPPPRQSPRREKALERAEGSATSTSTGGGEGRRDGERRLLVYGDGSTPQGALQAAGALLRHPPVVHDPESPAQRWLDDVAKLVMTARQRLDAGGRSSATKASGAATTGSASSRRRARRAAAAVRHSAATPSSTPSTREDLRGGPDARTSIERRRNDRRAAHATEGASSSRVSPQHGRGNQPSVPPVGGVGCRAFVASLRNVRWPPRFRPTIAEKYDGSVNPAEFLQVYTTGIEAAGGDDRVMANFFPMALKGQARGWLMNLPPASVHSWEDLCQQFTMNFQGTYPRPGEEADLHAVQRRDDESLRSYIQRFCQVRNTIPCIPAHAVIYAFRGGVRHNRMLEKIASKEPQTTAELFQLADRVARKEEAWTWNPSGSGVAASAAPGSAAQTGRRDRRRKKRSAHTDDEGHVLAVEGAPRATRKGRPAGDKKKEAGAPSRERPTGKWCTVHNTSLHDLADCRVVTDRSDRGLGGHRRSDTRLTPSRQDEGF

>LOC_Os03g53130.1 Accession ID: AAR87302.1

MSNKVTFQIVHGEGNIRFGPDCVDLSDFVMTSKGIDRPVERTFQSIYSWLLRGFRIDQEVYTMSVSVVVSRATESYFWELMPMDSTAAWRRYVEMAFERSWPLVIFVSVQEKDINVSMQTEDVEGPINAGDVVGPSMQNEENQPREEQAMGMADEGERVGIIVDEMEREDSDNEEADDDASSDEEGDVMATDWANEDFSRLVISEGDHVPWEYKENEVIEGARYAHKDEMKEAVKHWAVSLQREFRVVKSTNYVYEVRCMKEDCPWRVHAYKGKWNDYWKVSIVTEHKCYLQGVEKYHRNITSAFVASEMYSSVVGNIGFEPKSIIRHIENKFKYTISYAKAWRAKQKIIEMRYGTFEASYDNLPRLLATIAQRNNNTYYDLHTFTSVDDRTKSVLQRAFFSLGACINAFVHCRPVLCIDGTFMTGKYRGQILTAIGCDGNNQVLPMAFAFVESENTESWYWFLERVHIAVVRMRPNVCLIHDRHAGMLRAIDYLQNGWDEKGLPPKWPDVRSRWCMRHMGANFYKQFKNKHLMELFKRLCAQNQEKKFNELWDKLDELTTKQTDEQSRRPQVEGDEPPIPLGALHDDPPTMRRRSGSSIRNFTQWIENEPKEKWSLLFDTDGSRYGIMTTNLAEVYNWVMRGVRVLPLVVIVEFILHGTQAYFRDRYKKIGPSMADNNIVFGNVVTKYMEDKIKKARRHRVVAQGTQVHRYEIMCVDRSRRGIYRKQAVQECVLKADGGCTCSCMKPKLHHLPCSHILAAAGDCGISPNVYVSNYFRKEAIFHTWSEEIYGFGISGSYTTLSAQVFYVPDPSKLRVKKGRRQTRRIRNDMDESEAGGRTLRCSKCDLRGHTYKKCPKNAEVPSGADASPSGHSSDGMAYDTPALLNRGIDRNHRSFLSAVEGAQLGTFRPRTSREWLRVDPRHVPWLRAAGLLPLCRLIEAAADDRDPAKRWDADRSLLAALVDRWRPETHTFHLPCGEMAPTLQDVSYLLGLPLAGAPVGPVDGVFGWKEDITARFEQADLLHPDADDYSVRRSLEAYLLWLFEWVMFTSTHGHAVDFRLVHYAAERFAIGRPVVDSAPYGVGRSAQWPEDGPTMGTYWCRRGRRYAHVQVRRGYPDLVFEFDRLQPSDVIWEPYTEEAVAARAPLGLSSLCTRDQAYWLTILPMVFDIFVEPHCPQRVMRQFGLRQLAGALWAPRVQQYVDDWVLATEEVINELFPHTEENYRDYLRWYLPRTRARVTFTPDAPEPHVAAVTDAYPTHRDRDYFVGADAARDISADITAVQVRLNRGLHLTDVEQRVTFDRMQEKMRAVMRVFSCRSAVDVVPPAGPVQPRPRAPTVGAGPRPTAPVSHGPRLPSSAFAGTTGASASSAGAFATSSGAFASSSSHGAPIPRPHAGFAAGIFGTGASSSHAGRTGPTSQFYDDDLHGAHHHDVLGSSQLGGAPEAHTQEQPEVTPVQAGRVGRAVPPDRLTYSQGHIRAQGRRDRGKRPRQ

>LOC_Os08g11160.1 Accession ID: ABA97888.1

MGVAGESMPLVVEGVIMIQERCTVWYTSGQSKTIRIAVSAVMVCVPRLIPGRGFYAQISSEITSEFLGVTTHQWGFGYSRVNKAIIAITIIIKS

>LOC_Os10g24590.1 Accession ID: ABB47444.1

MEVDEVGGRWTFSRANEGYTSCGPVVEMSWHRAGVLLLGAQSCLPVPGVPAVDGIGSVLLSMARMGWKLCHVFRLYTVVMAGSVSRRGLDMTGFVLELRGMCVVLGYPHGVDYQARPFPEQEGDDAEPHAAWEVTAVILAGSPERTSLTVTAGGDSFPAACQSAALLAIGTLHQRYPDELQHSPYRYHPRRGGARDYATFRDASSEDDATIVHLARMVEAYDAARIDFHQMVRRGMVENNMKILELRQENLQLKKDLDAVEAQLHQLKIAQGEICRPKHRRVYRSQKITARKSTSRPELVRQSLAWTCFVETPRAEPAPVVPQEGEASGVGSTEDALLLTFRPGPSQQ

>LOC_Os03g37800.1 Accession ID: AAP12960.1

MKIGDSSNSGAAVERVIANSGAVELPLLTRTNYHEWSLVMRVSLEALGLWDTVEKDKLEHREGRARCEEVAKEARAAVEKMRVGDDRAKSASMQRLLKEFKNVAFHDGESVDDFAMHINGLVASLAQAWRGNGGQPCGEEDPCAWSPIR

>LOC_Os12g36480.1 Accession ID: ABA98908.2

MNENENDRTNESENENDRTKTSENEGTNVNENKRTNVNENERTNVNELGNMADRDEEQILYDTIVEGSSQYWNEEEGNEDPNQYLNEKGNVERDAEGNQQGHVERDVEGNQEEEASGSQPSVEQKRARGQRGAAKKLEGRHIITEVEEDGRPSAPAEAAKNYVRHSGWVVRDNVPVSTVYWRRTRARGDHESFVPDSEKEMLWTTMLETFTLPAGTEDKVKRWTLKKMAEQFQSFKGDLYQKYILKGQTPNFDTFPKLRDHWDEFVAYKTGEQGQAMMERNKENAAKKKYHHHLGSGGYSVAMPKWEQMEARLIERGIEPATANWPERSKFWYYAHGGTLNPADGSQVFGYQIQEAARRLTDAVEASSQGTFRPDRDRDELTLALQTPEHPGRTQGKGVIPWKIGFKEDIHTYRSRMRSKRDTEAKIADLEFRVSSYELNMQEEVARKVDERMAAHRSHDPQPTIPPAMVSPSGNRSSCASTGQVGSQSMDAIQTQDESTCPVDDITQRTPCELHIPFKNLSIKVASGMAIPTDPSGTYHCRPIPAGYSKVEVELVEGAYEDLELDYPGGDAPSPPQAPASTPPQDPAPTPPRAPTPTPPQAPLPAPSKSRATPTPPLAHTRATKKAKVDAAKNKDPGYDCTQEELDAYVASEVKRQFKPRSPEKKIPIDPSVRNFFRGISASVKEAIKLSDYERTLKKASSGKSKPVPQLGEQPNQEIEPLVTGKEMTIEQFITDTGLTTDQLLGVAPIEKAEVKYMYELGKPLVKPELLQSLPTQMYKFHQLMEMSATGREMIGARIRDTDFLQGDDILWINFRGIYELYQLDALDVSIISCWILMEIQRARRRVFDTGFIDPRKVNVAMLDQYPQETEDNLVHLLKAQHYKTFILLPYNTEFHWVLLLIDLEACTVNVYDSMDKKESTFDKCAKQKQGTNLCGYYVCEYCHCLADQIITTRELDFIRMRDNLTTHKEFIAAVQEQLMGFINEEILDPKGEFYYDGNTIHRSLASELLRRNRS

>LOC_Os06g35310.1 Accession ID: ABA96994.1

MGQRLGQMGLALVRPNPGLDKESAFYLDITVGGSLMHKTPSEGRTILDHILENTSFMTQSDEPQPEAYVSKIEEPLTIEPLTEPSTSASSIDEKVAKQPSVENEEIQTPDRAAILFRDDFNEDYGNTLNYFSKKKSLVPLPPPDPMELGFLRETVQS
